# Humoral immunity prevents clinical malaria during *Plasmodium* relapses without eliminating gametocytes

**DOI:** 10.1371/journal.ppat.1007974

**Published:** 2019-09-19

**Authors:** Chester J. Joyner, Cristiana F. A. Brito, Celia L. Saney, Regina Joice Cordy, Maren L. Smith, Stacey A. Lapp, Monica Cabrera-Mora, Shuya Kyu, Nicolas Lackman, Mustafa V. Nural, Jeremy D. DeBarry, the MaHPIC Consortium, Jessica C. Kissinger, Mark P. Styczynski, F. Eun-Hyung Lee, Tracey J. Lamb, Mary R. Galinski

**Affiliations:** 1 Malaria Host–Pathogen Interaction Center, Emory Vaccine Center, Yerkes National Primate Research Center, Emory University, Atlanta, GA, United States of America; 2 Division of Pulmonary, Allergy, Critical Care, & Sleep Medicine, Department of Medicine, Emory University, Atlanta, GA, United States of America; 3 Laboratory of Malaria, Centro de Pesquisas René Rachou–Fiocruz, Belo Horizonte, MG, Brazil; 4 Department of Biology, Wake Forest University, Winston-Salem, North Carolina, United States of America; 5 School of Chemical & Biomolecular Engineering, Georgia Institute of Technology, Atlanta, GA, United States of America; 6 Institute of Bioinformatics, University of Georgia, Athens, GA, United States of America; 7 Department of Genetics, University of Georgia, Athens, GA, United States of America; 8 Center for Tropical and Emerging Global Diseases, University of Georgia, Athens, GA, United States of America; 9 Lowance Center for Human Immunology, Emory University, Atlanta, GA, United States of America; 10 Division of Microbiology and Immunology, Department of Pathology, University of Utah, Salt Lake City, UT, United States of America; 11 Division of Infectious Diseases, Department of Medicine, Emory University School of Medicine, Atlanta, GA, United States of America; McGill University, CANADA

## Abstract

*Plasmodium* relapses are attributed to the activation of dormant liver-stage parasites and are responsible for a significant number of recurring malaria blood-stage infections. While characteristic of human infections caused by *P*. *vivax* and *P*. *ovale*, their relative contribution to malaria disease burden and transmission remains poorly understood. This is largely because it is difficult to identify ‘bona fide’ relapse infections due to ongoing transmission in most endemic areas. Here, we use the *P*. *cynomolgi*–rhesus macaque model of relapsing malaria to demonstrate that clinical immunity can form after a single sporozoite-initiated blood-stage infection and prevent illness during relapses and homologous reinfections. By integrating data from whole blood RNA-sequencing, flow cytometry, *P*. *cynomolgi*-specific ELISAs, and opsonic phagocytosis assays, we demonstrate that this immunity is associated with a rapid recall response by memory B cells that expand and produce anti-parasite IgG1 that can mediate parasite clearance of relapsing parasites. The reduction in parasitemia during relapses was mirrored by a reduction in the total number of circulating gametocytes, but importantly, the cumulative proportion of gametocytes increased during relapses. Overall, this study reveals that *P*. *cynomolgi* relapse infections can be clinically silent in macaques due to rapid memory B cell responses that help to clear asexual-stage parasites but still carry gametocytes.

## Author summary

*Plasmodium vivax* contributes significantly to global malaria morbidity and remains a major obstacle for malaria elimination due to its ability to form dormant stages in the liver. These forms can become activated to cause relapsing blood-stage infections. Relapses remain poorly understood because it is difficult to verify whether *P*. *vivax* blood-stage infections in patients are due to new infections or relapses in most cases. Here, we use a nonhuman primate model of *Plasmodium vivax* malaria in concert with state-of-the-art immunological and molecular techniques to assess pathogenesis, host responses, and circulating gametocyte levels during relapses. We found that relapses were clinically silent compared to initial infections, and they were associated with a robust memory B cell response. This response resulted in the production of antibodies that were able to mediate clearance of asexual parasites. Despite this rapid immune protection, the sexual-stage gametocytes continued to circulate. Our study provides mechanistic insights into the host-parasite interface during *Plasmodium* relapse infections and demonstrates that clinically silent relapses can harbor gametocytes that may be infectious to mosquitoes.

## Introduction

Due to their ability to establish dormant forms in the liver called hypnozoites, relapsing malaria parasites pose a significant obstacle to malaria elimination [1]. Hypnozoites can become activated, resulting in repeat blood-stage infections, and these relapses account for the majority of *P*. *vivax* blood-stage parasitemias [2, 3]. This raises critical questions about the relative importance of relapses in causing illness, and their importance in transmission. However, there are very few studies that have directly examined relapse biology, especially in the context of pathogenesis, host immunity, and transmission. An improved understanding of relapses and their immunological and epidemiological implications is needed to design effective control and elimination strategies for relapsing malaria parasites.

There are no rodent malaria parasites species that form hypnozoites, making these models inadequate for studying relapses. Human studies in endemic areas have limited utility because it is generally difficult to determine whether a blood-stage infection resulted from new, relapsing, or recrudescent infections [4, 5]. Although approaches such as parasite genotyping, relocation of individuals from *P*. *vivax* endemic areas to non-endemic areas, and mass drug administration provide more confidence that a *P*. *vivax* infection is due to a relapse, these approaches have caveats [6–10]. For example, new genotypes detected in sequential blood-stage infections could be due to the activation of hypnozoites that did not originally activate and circulate in the blood at the time of an initial sample collection; thus, these parasites could be mistaken as parasites from a new infection even if they originated from hypnozoites in the liver. Additionally, the inability to control for an individual’s infection history complicates the investigation of immune responses during human relapse infections.

Nonhuman primate (NHP) models lack these barriers and present several advantages for studying relapses. In particular, rhesus macaques infected with *Plasmodium cynomolgi*, a simian malaria parasite closely related to *P*. *vivax*, recapitulate the biological, clinical and pathological features of *P*. *vivax* malaria, including hypnozoite formation and relapses [11–15]. Further, *P*. *cynomolgi* is now recognized as a zoonotic species in South East Asia, making understanding its biology a priority [16–18]. These species have similar 48-hour intraerythrocytic developmental cycles, antigenic makeup, and infected red blood cell (iRBC) modifications that include abundant caveolae vesicle complexes [19–22]. Moreover, genomes and immunological tools are available to support the study of host-parasite interactions and NHP immune responses. Hence, these factors make the rhesus macaque–*P*. *cynomolgi* model valuable for the study of relapse biology.

We previously showed that *P*. *cynomolgi* relapses in rhesus macaques have substantially reduced parasitemias compared to primary infections, and they do not result in anemia or other clinically detectable disease manifestations [12]. The present study explored the host-pathogen interactions that underpin clinically silent *P*. *cynomolgi* relapses to garner insights into asymptomatic *P*. *vivax* relapses in humans [7, 23, 24]. We hypothesized that humoral immunity could explain the lack of clinical disease during relapses since passive transfer of antibodies has been shown to control parasitemia and ameliorate disease during human, NHP, and rodent *Plasmodium* infections [25–27]. Here, we demonstrate that lack of clinically detectable disease during *P*. *cynomolgi* relapses is associated with rapid memory B cell responses and the swift rise of anti-parasite IgG1 antibodies that can mediate clearance. The same humoral immune responses were also associated with protection against subsequent challenge with the same parasite strain about 60 days after radical cure. Interestingly, the immune response reduced the number of sexual stage gametocytes present compared to the primary infection, but the cumulative proportion of gametocytes increased during relapses. This suggests that the immune response generated by the infection primarily targeted the asexual stages. Concordantly, mature gametocyte gene expression was not significantly different between primary infections and relapses. Together, these data show that clinically silent, *P*. *cynomolgi* relapses carry gametocytes despite a significant reduction in parasitemia associated with an effective humoral immune response. Overall, this study broadens our understanding of relapsing malaria parasite pathogenesis and infections, with important epidemiological implications relevant to malaria elimination strategies.

## Results

### Relapses are clinically silent and do not cause inflammation

**Fig 1:**
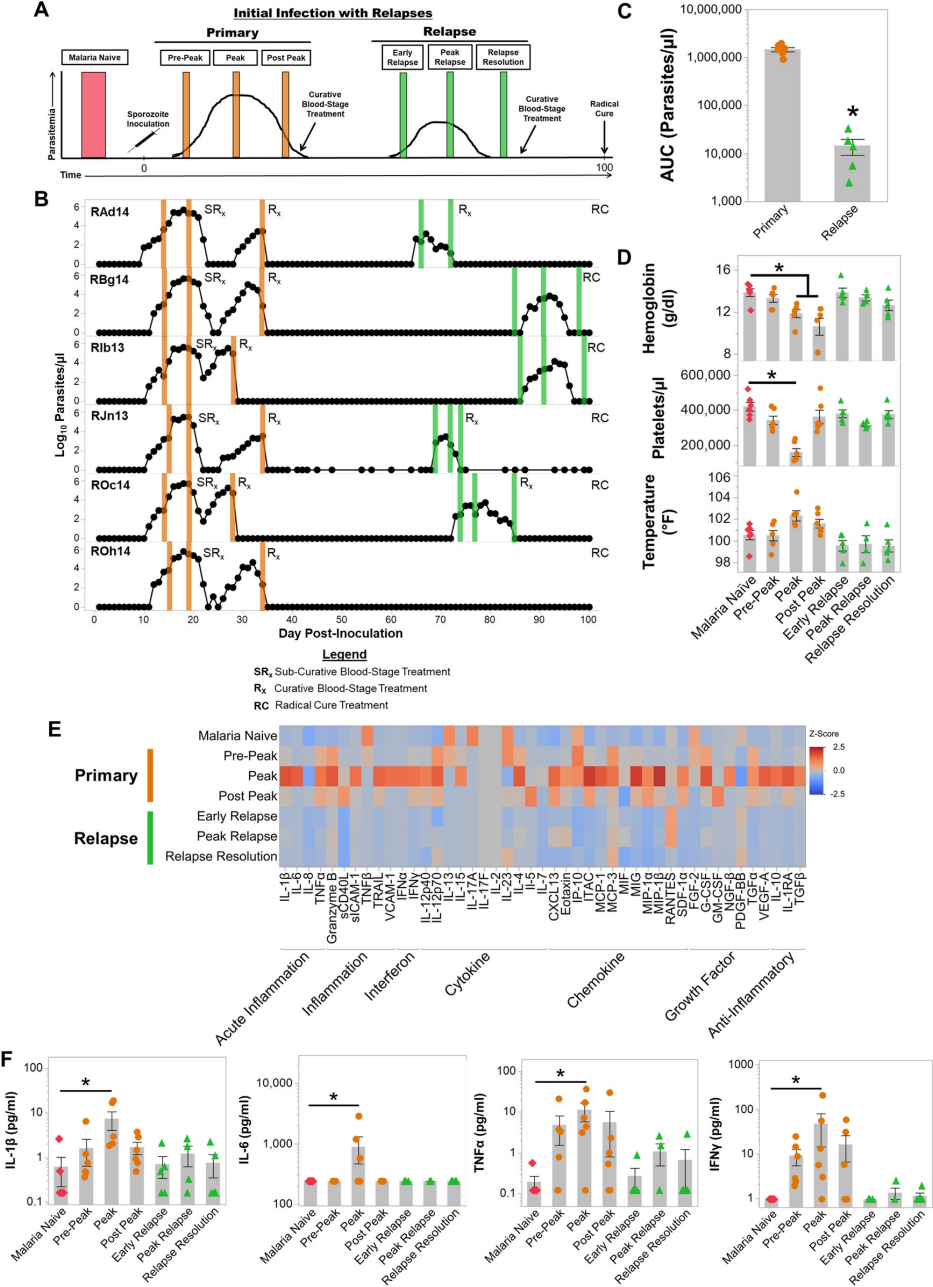
*Plasmodium cynomolgi* relapses do not cause illness in rhesus macaques infected with *Plasmodium cynomolgi* M/B strain. (a) Idealized study design for collecting blood specimens at different points during initial primary infections and relapses in rhesus macaques infected with *P*. *cynomolgi* M/B strain. (b) Parasite kinetics over a 100-day *P*. *cynomolgi* M/B strain infection in rhesus macaques as determined by Giemsa-stained blood films. Each row represents a different individual. Colored bars indicate when sample collections were performed during initial infections (orange) or relapses (green). These collections correspond from left to right with the idealized experimental schematic in panel a. (c) Cumulative parasitemia as determined by area under the curve analysis during initial infections and relapses. (d) Mean hemoglobin levels, platelet concentrations, and temperature prior to infection (pink diamonds), during the initial infections (orange circles) and during relapses (green triangles). Gray bars are the mean of the individual data points. (e) Heatmap of z-score transformed cytokine, chemokine, and growth factor concentrations at each infection point as determined by a multiplex cytokine array. (f) Plasma concentrations of selected cytokines during primary and relapse infections. Statistical significance was assessed by a linear mixed-effect model with a Tukey-Kramer HSD post-hoc analysis for all analyses. Asterisks indicate p< 0.05 in comparison to the malaria naïve condition unless otherwise indicated. Error Bars = SEM.

Six rhesus macaques were inoculated intravenously with 2,000 *P*. *cynomolgi* M/B strain sporozoites on Day 0, and the parasitemia and clinical status of each individual monkey was evaluated daily for up to 100 days by light microscopy and complete blood count (CBC) analysis, respectively. Blood specimen collection time points are indicated in Fig 1A and 1B. A summary of the clinical and parasitological criteria for each specimen collection is provided in S1 Table.

The infections reached patency between days 10–12 (mean ± SE = 11.16 ± 0.3) after inoculation. Parasitemia peaked between 276,981–540,156 parasites per microliter of blood (mean ± SE = 400,563 ± 33,028 parasites/μl) between days 17–19 post-inoculation (Fig 1B). After collecting blood samples at the peak, all animals were administered a sub-curative dose of artemether to reduce but not eliminate the blood-stage parasites. This treatment ensured that the animals would remain parasitemic but not develop severe disease. Following the administration of curative blood-stage treatment, relapses were observed at different time intervals for each individual (Fig 1B). As anticipated based on earlier studies, relapse infections had approximately 200-fold lower parasitemia than the primary infections (p < 0.05; Fig 1B and 1C). Notably, relapse parasitemias declined below patency 7 to 15 days after their detection in the blood without treatment (Fig 1B).

The primary infection induced a myriad of clinical manifestations with elevated temperatures (mean ± SE = 102.3 ± 0.44°F) although not statistically significant and was accompanied by significant decreases in hemoglobin levels and platelet counts compared to when the animals were naïve (Fig 1D). Temperatures remained elevated post-peak for approximately one week after the administration of subcurative artemether treatment (Fig 1B and 1D). The anemia severity was moderate to severe with hemoglobin nadirs ranging from 5.7–9.0 g/dl (mean ± SE = 7.3 ± 0.4 g/dl) after the peak of parasitemia (S1A Fig). Platelet counts dropped from a mean of 421,250 platelets/μl when naïve to an average of 119,000 platelets/μl at the peak of the primary infection (Figs 1D and S1B). In stark contrast to the primary infections, anemia, thrombocytopenia, and fever were not observed in the animals during relapses (Fig 1D, S1A and S1B Fig).

Clinical severity of malaria has been correlated with inflammatory responses to infecting *Plasmodium* parasites [28–31]. Congruent with elevated temperatures and clinical presentation, inflammation was highest at the peak of parasitemia when 22 out of 45 cytokines, chemokines, or growth factors tested were significantly increased in the plasma compared to malaria naïve values (Figs 1E, 1F and S2). Pyrogenic cytokines such as IL-1β, TNFα, IL-6 and IFNγ were significantly increased in plasma at peak parasitemia when the animals were presenting with clinical illness (Fig 1F). Notably, only 6 out of 45 analytes remained significantly elevated after the administration of sub-curative artemether treatment (S2 Fig). In concordance with the clinical presentations, cytokine responses were subdued during relapses compared to the primary infection (Fig 1E). IL-1β, TNFα, IL-6 and IFNγ were not significantly elevated during relapses compared to pre-infection values, and Monokine Induced by Interferon Gamma (MIG) was the only cytokine significantly increased during relapses (Figs 1F and S2). The decrease in inflammation was likely due to the significant reduction in parasitemia that was observed between the initial infections and relapses (Fig 1B and 1C). Overall, these results reaffirmed that *P*. *cynomolgi* relapses had substantially reduced parasitemia and did not cause clinical signs of malaria.

### Transcriptional profiling identifies significant changes related to B cells during relapses

To identify host responses that may reduce parasitemia and disease during relapses, we performed RNA-Seq on whole blood samples collected at the time points indicated in Fig 1A. The sequencing reads were mapped to concatenated host and parasite genomes, normalized by library size, log2 transformed, and finally, variance due to inter-individual variability was removed via the SNM transformation as previously described [32, 33].

**Fig 2:**
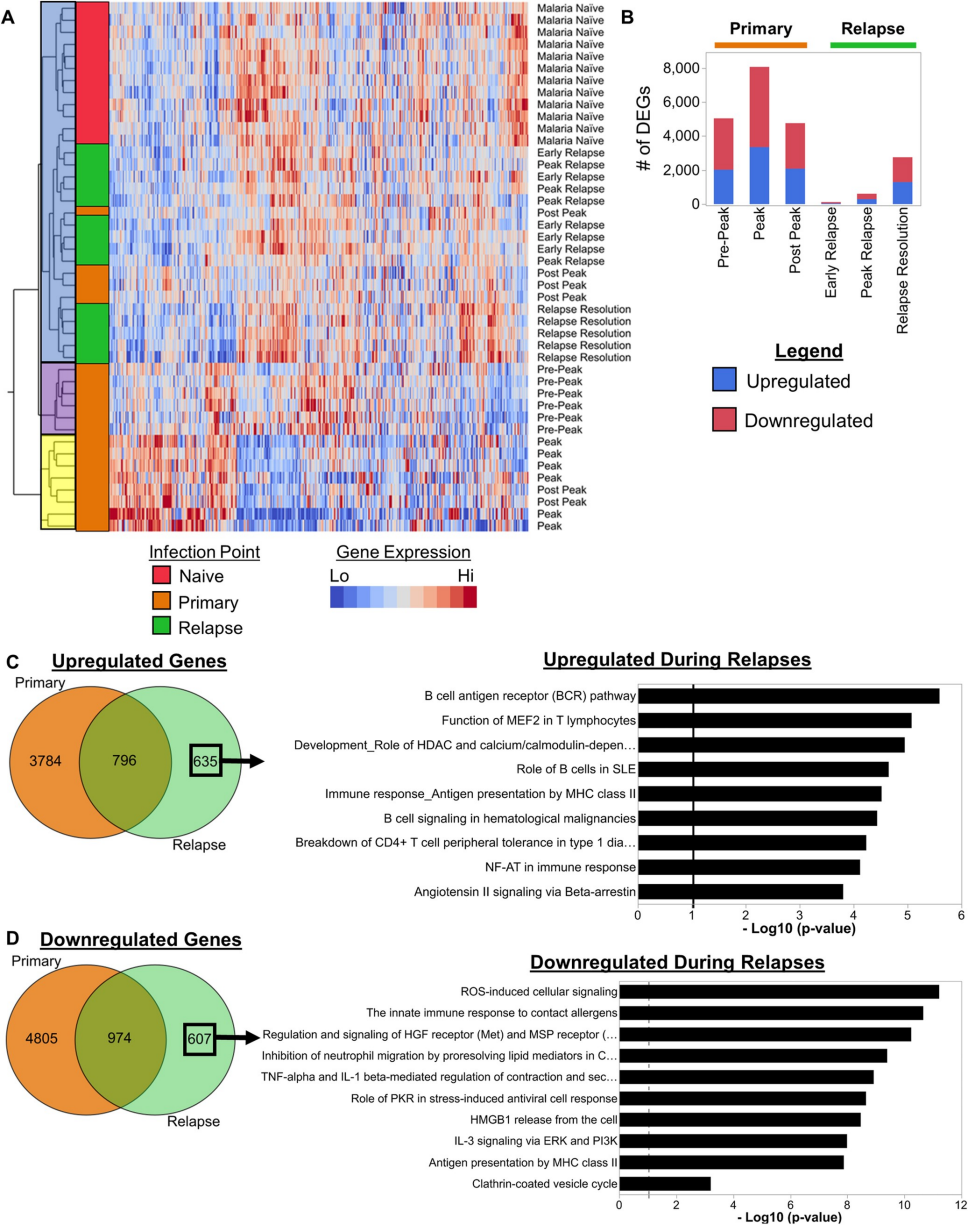
*Plasmodium cynomolgi* relapses cause distinct changes in host transcriptomes related to B cells. (a) Hierarchical clustering using Ward’s method of whole blood transcriptomes at different infection points during *P*. *cynomolgi* M/B infections of rhesus macaques. (b) The number of differentially expressed genes identified at each infection point during primary and relapse infections. (c) Venn diagram showing the overlap of upregulated genes during primary infections and relapses. The upregulated gene pathways identified using the upregulated genes unique to relapses are shown. The solid black line in the pathway plots indicate an FDR corrected p-value of less than 0.05. (d) Venn diagram showing the overlap of downregulated genes during primary infections and relapses. The top 10 downregulated gene pathways identified using the downregulated genes unique to relapses are shown. The solid black line in the pathway plots indicates an FDR corrected p-value of less than 0.05.

Unsupervised hierarchical clustering of transcriptional profiles identified three major clusters as indicated by blue, purple, and yellow shading; these clusters captured approximately 30% of the variance associated with changes in gene expression (Fig 2A). Generally, the clinical presentation appears to drive the clustering pattern. The blue cluster consists of samples collected when the animals were not experiencing clinical symptoms, including prior to infection, post peak when the animals were recovering from illness, and during relapses (Fig 2A). In contrast, the yellow cluster consists of samples collected at the peak when clinical signs of malaria were evident, and the purple cluster consists of samples acquired after parasites were detected in the blood but prior to the onset of clinically detectable disease (Fig 2A). These results demonstrated that distinct changes in the host transcriptome occurred during the course of *P*. *cynomolgi* blood-stage infections. Samples collected as relapses were resolving (i.e. relapse resolution) formed a distinct subcluster compared to the early and peak relapse samples within the blue cluster (Fig 2A). Importantly, this subclustering showed that the transcriptional changes associated with resolving relapse infections were similar across individuals and distinct from the transcriptional changes during primary infections.

In agreement with the clustering pattern, differential gene expression analysis identified major transcriptional changes during the primary infection with more muted changes during relapses. Differentially expressed genes (DEGs) were determined via ANOVA followed by a t-test post-hoc analysis with Benjamini-Hochberg false discovery rate (FDR) correction. Genes with an FDR adjusted p-value of less than 0.05 were considered significantly differentially expressed. The DEG analysis was focused on the identification of genes that are differentially expressed between the malaria naïve time point and each subsequent time point (e.g. malaria naïve vs. peak, malaria naïve vs. relapse resolution, etc.). Compared to when the animals were naïve, the largest number of DEGs were identified during the peak of parasitemia, with approximately 3,350 and 4,713 DEGs upregulated and downregulated, respectively (Fig 2B). The pre-peak and post-peak time points induced comparable changes in host gene expression with over 2,000 genes upregulated and over 2,000 genes downregulated for each (Fig 2B). In contrast, relapses caused substantially less changes in host-gene expression compared to when the animals were naive (Fig 2B). Only 44 upregulated and 74 downregulated DEGs were identified during the early relapses, and 291 upregulated and 316 downregulated DEGs during the relapse peaks (Fig 2B). In contrast, the relapse resolution infection points had the most DEGs during relapses with 1,294 upregulated and 1,459 downregulated DEGs (Fig 2B).

Next, we focused on genes that were only differentially expressed during relapses. All upregulated and downregulated DEGs during the primary and relapse infections were compared. Six hundred and thirty-five upregulated and 607 downregulated DEGs were determined to be unique to relapses (Fig 2C and 2D). Metacore pathway enrichment analysis based on these upregulated and downregulated DEG sets revealed nine and over 50 significantly enriched pathways, respectively (S2 and S3 Tables). The nine upregulated pathways identified during the relapses are related to B cells, T cells, cell signaling, and antigen presentation (Fig 2C). The pathway with the highest enrichment score was related to B cells, and three out of the nine pathways are related to B cell responses (e.g., B cell responses in SLE, B cell signaling in hematological malignancies, and the B cell receptor pathway) (Fig 2C). The genes that were responsible for the enrichment of these pathways are composed of B cell surface proteins such as the BAFF-R, CD79A, and CD79B in addition to signaling molecules like Btk and VAV-2 (S2 Table). In contrast, the pathways related to T cells, signaling, and antigen presentation are composed of genes such as Akt, MAP kinases, CD86, T-bet, MHC-II, etc., which are expressed by a variety of immune cells, including B cells (S2 Table). The top downregulated DEGs during relapses belong to pathways related to inflammation, cell damage responses, and innate immune cells, such as dendritic cells, macrophages and neutrophils (Fig 2D and S3 Table).

Together, this analysis showed that relapses induced relatively minor, but unique changes in the host transcriptome compared to the primary infections that were predominantly related to the downregulation of pathways involved in innate immune responses and upregulation of pathways related to B cells.

### Switched and unswitched memory B cells expand during relapses

**Fig 3:**
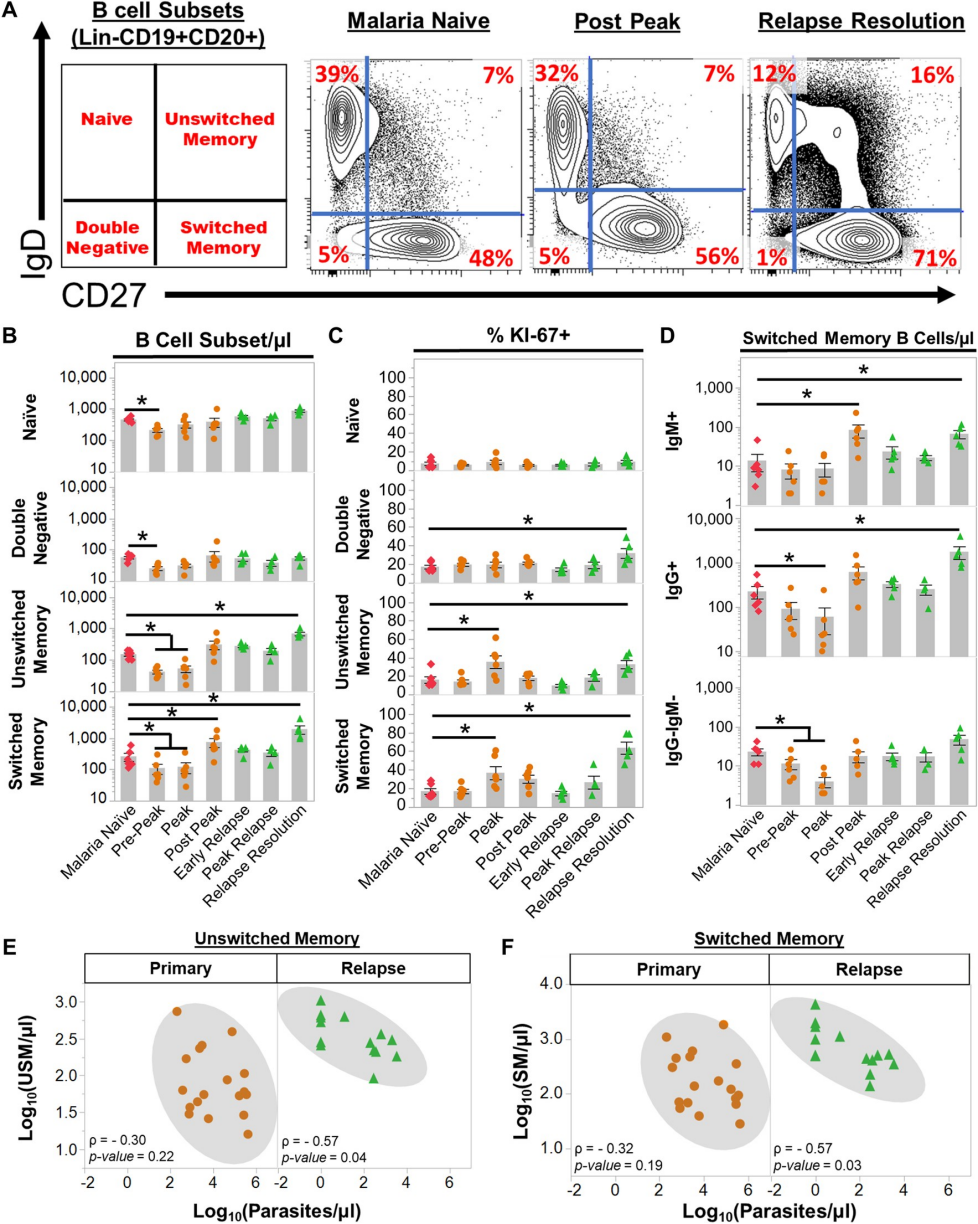
*Plasmodium cynomolgi* relapses induce an expansion of unswitched and switched memory B cells. (a) The frequencies of four peripheral blood B cell subsets in rhesus macaques as determined by flow cytometry prior to infection (Malaria Naïve), after the peak of parasitemia during the initial infection (Post Peak), and during the resolution of a relapse (Relapse Resolution). A representative sample is shown. The far left panel indicates the B cell subset in each quadrant based on the gating strategy in S3 Fig. Red numbers in each quadrants indicate the percentage of each subset out of Lin-CD19+CD20+ B cells. (b) Absolute numbers of naïve, double negative, unswitched memory, and switched memory B cells at each infection point. (c) The percentage of each B cell subset that is KI67+ at each infection point as determined by flow cytometry. (d) The absolute number of IgG+, IgM+, and IgG-IgM- switched memory B cells during initial infections and relapses as determined by flow cytometry. (e) Spearman’s correlation analysis of the number of unswitched memory B cells and parasitemia during primary infections and relapses. (f) Spearman’s correlation analysis of the number of switched memory B cells and parasitemia during primary infections and relapses. Pink diamonds = malaria naïve, orange circles = initial infection, and green triangles = relapse infections. Gray bars indicate the mean of the data points shown; Error Bars = SEM. Statistical significance was assessed by a linear mixed effect model using a Tukey-Kramer HSD post-hoc analysis. Asterisks indicate a p-value < 0.05. ρ = Spearman’s correlation coefficient.

Since the transcriptome data suggested that B cell responses may be involved in ameliorating disease during relapses, we next performed flow cytometry analysis on peripheral blood mononuclear cells (PBMCs) isolated during the primary and relapse infections. We utilized a B cell immunophenotyping strategy previously developed for human immunology studies and optimized it for specimens collected from rhesus macaques [34]. With this strategy, B cell subsets in PBMCs are CD19 and CD20 positive and further classified into four subsets based on the surface expression of IgD and CD27 (Figs 3A and S3). Similar to humans, four B cell subpopulations were identified in PBMCs from rhesus macaques: naïve (IgD+CD27-), unswitched memory (USM: IgD+CD27+), switched memory (SM: IgD-CD27+), and double-negative B cells (DN: IgD-CD27-). Surface IgM was present in all subsets, as previously shown for humans, albeit at low frequencies in the SM compartment (S3B and S3C Fig; [34, 35]). IgG surface staining was evident in the SM and DN populations but not in naïve B cells (S3B and S3C Fig). In summary, this immunophenotyping strategy yielded comparable results for samples acquired from either humans or rhesus macaques.

All B cell subsets decreased in the blood at pre-peak (Fig 3B). This decrease was consistent with the pan-lymphopenia observed during the initial infection (S4 Fig). At the peak, naïve and DN B cells stabilized in the periphery whereas SM and USM B cells remained significantly reduced compared to when the animals were naive (Fig 3B). Although USM and SM B cell numbers were reduced, the percentage of Ki67+ USM and SM B cells was significantly increased at the peak, suggesting these cells were activated (Fig 3C). Following subcurative treatment, USM B cells stabilized and SM B cells significantly increased from 263 ± 75 cells/μl (mean ± SE) when the animals were uninfected to 761 ± 242/μl (mean ± SE) post-peak (Fig 3B). During relapses, USM B cell numbers expanded from 153 ± 187/ μl (mean ± SE) when naïve to 686 ± 89/μl (mean ± SE), and SM B cells rose to 1982 ± 590 cells/μl (mean ± SE) (Fig 3B). The increase in the absolute numbers was accompanied by an increase in the frequency of Ki67+ USM and SM B cells, consistent with a new expansion of these cells during the relapses (Fig 3C). In contrast, the absolute numbers of naïve and DN B cells were unchanged (Fig 3B and 3C). Notably, there was an increase in frequency of Ki67+ DN B cells during relapses, although the absolute numbers of this population did not increase (Fig 3B and 3C).

Parasite-specific IgG and IgM are important for controlling parasitemia. Early during relapses IgG+ SM B cell frequencies were 330 ± 49/μl, which is similar to the naïve numbers (225 ± 71/μl), but these rapidly increased approximately 8-fold to 1,768 ± 567/μl as the relapse infections resolved (Fig 3D). Thus, the expansion in IgG+ SM B cell numbers was induced by the relapse. IgM+ SM B cells also increased in response to the relapses, but the absolute numbers of these cells were smaller in comparison to the IgG+ SM B cells (Fig 3D).

Finally, we evaluated if the changes in B cell subsets were correlated with parasitemia during primary and relapse infections. There was not a significant correlation between the number of naïve, DN, USM, and SM B cells and parasitemia during the primary infections (Figs 3E and 3F and S5). In contrast, USM (Spearman’s ρ = -0.57, p = 0.04) and SM (Spearman’s ρ = -0.57, p = 0.03) B cells were inversely correlated with parasitemia during relapses (Fig 3E and 3F). Notably, naïve and DN B cell numbers were not inversely correlated with parasitemia during relapses (S5 Fig). Overall, these data illustrate that USM and SM B cells dramatically expand during relapses in response to a new blood-stage parasitemia and may be involved in controlling parasitemia and ameliorating disease.

### Anti-parasite IgG1 increases during relapses and opsonizes infected RBCs for phagocytosis

**Fig 4:**
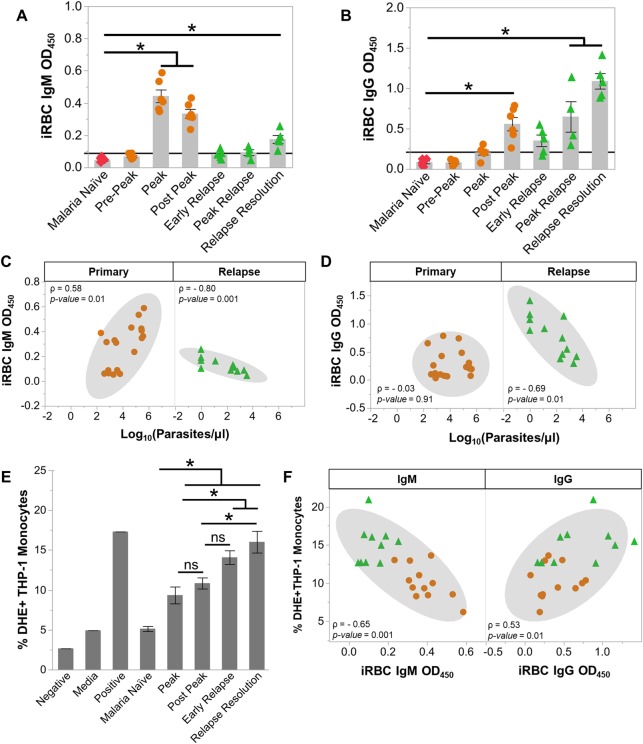
Anti-parasite IgG1 is produced rapidly during relapses and opsonizes *P*. *cynomolgi* infected RBCs. Kinetics of anti-iRBC IgM (a) and anti-iRBC IgG (b) response during primary and relapses infections as determined by ELISA; the black line indicates background defined as the average of the malaria naïve time point plus three standard deviations. Spearman’s correlation analysis of anti-iRBC IgM (c) and anti-iRBC IgG (d) responses in relation to parasitemia during primary or relapse infections. (e) Percentage of THP-1 monocytes that phagocytosed *P*. *cynomolgi* M/B strain iRBCs after opsonization with heat-inactivated plasma collected at different infection points during initial infections and relapses. (f) Spearman's correlation analysis of opsonic phagocytosis activity and anti-iRBC IgM and IgG during primary infections and relapses. Gray bars indicate the mean of the data points shown; Error Bars = SEM. Statistical significance was assessed by a linear mixed effect model using a Tukey-Kramer HSD post-hoc analysis. Asterisks indicate a p-value < 0.05. ρ = Spearman’s correlation coefficient.

Since B cell responses and antibodies are critical for suppressing parasitemia, we determined whether anti-parasite IgM and IgG were increased during relapses using an ELISA with infected RBC (iRBC) and uninfected RBC (uRBC) lysates. Total IgM (tIgM) increased from 0.34 ± 0.02 mg/ml to 1.93 ± 0.18 mg/ml (mean ± SE) at the peak of the primary infections and remained elevated post-peak (S6A Fig). *P*. *cynomolgi*-specific IgM increased at the peak and remained increased post-peak (Fig 4A). Total and parasite-specific IgG also increased at post-peak (Figs 4B and S6B). The IgG subclass of the iRBC-specific IgG produced during the primary infections was IgG1 (S6C Fig). Neither IgM nor IgG were inversely correlated with parasitemia during the primary infections (Fig 4C and 4D).

Notably, we did not observe a difference between the reactivity of IgM that recognized iRBC versus uRBC lysates during the primary infections (S7A Fig). Similar to IgM, uRBC-specific IgG also increased during the primary infections, and a significant difference between IgG recognizing iRBC versus uRBC was also not discernable (S7B Fig). uRBC lysate-specific IgM and IgG were both inversely correlated with hemoglobin levels during the primary infections, suggesting that these antibody responses may be linked with the loss of uRBCs and the development of anemia during *P*. *cynomolgi* infections (S7C and S7D Fig) [36].

In contrast with the primary infections, relapses did not result in significant changes in tIgM levels (S6A Fig). However, there was an increase in iRBC-specific IgM at the relapse resolutions, but this increase was approximately half of what was detected during the primary infections (Fig 4A). In contrast, iRBC-specific IgG was rapidly produced during relapses and was significantly increased during the peak relapse and the relapse resolution periods (Fig 4B). These values are five-fold higher than those observed during the primary infections (Fig 4B). The increase in IgG occurred alongside the expansion of IgG+ SM B cells that peaked during relapse resolutions, strongly suggesting that this response was important for controlling parasitemia during a relapse (Figs 3D and 4B). As in the primary infections, the iRBC-specific IgG was IgG1 (S6C Fig).

Next, we determined if the iRBC specific IgM and IgG were inversely correlated with parasitemia during relapses. Notably, the early relapse time points for monkeys RBg14 and RIb13 were excluded from this analysis because these time points were taken one to two days before a relapse was patent. Including these time points would confound our analysis since the relapse resolution time points were also taken when the parasitemia was below patency. As expected, iRBC-specific IgM and IgG were inversely correlated with parasitemia during relapses (Fig 4C and 4D)

To determine the functionality of the antibodies generated during the primary and relapse infections with respect to clearance of iRBCs, we performed a phagocytosis assay using a THP-1 monocyte cell line, as previously described [37]. The percentage of THP-1 monocytes that phagocytosed iRBCs after being opsonized with heat-inactivated plasma increased from 5.1 ± 0.31% from naïve animals to 9.3 ± 1.1% and 10.8 ± 0.7% at the peak and post-peak of the initial infections, respectively (Fig 4E). During relapses, the percentage of THP-1 monocytes that phagocytosed iRBCs was even higher (16.0 ± 1.4%) (Fig 4E). Interestingly, the opsonization of iRBC by the heat-inactivated plasma was positively correlated with the amount of iRBC-specific IgG (Spearman’s ρ = 0.53, p = 0.01) and inversely correlated with iRBC-specific IgM (Spearman’s ρ = -0.65, p = 0.0017) (Fig 4F). Collectively, these data demonstrate that the anti-parasite antibodies produced during relapses can mediate iRBC clearance, consistent with a key role for IgG+ SM B cells in providing rapid host immunity to suppress parasitemia during relapses.

### Homologous reinfections after radical cure have low parasitemias and are clinically silent

**Fig 5:**
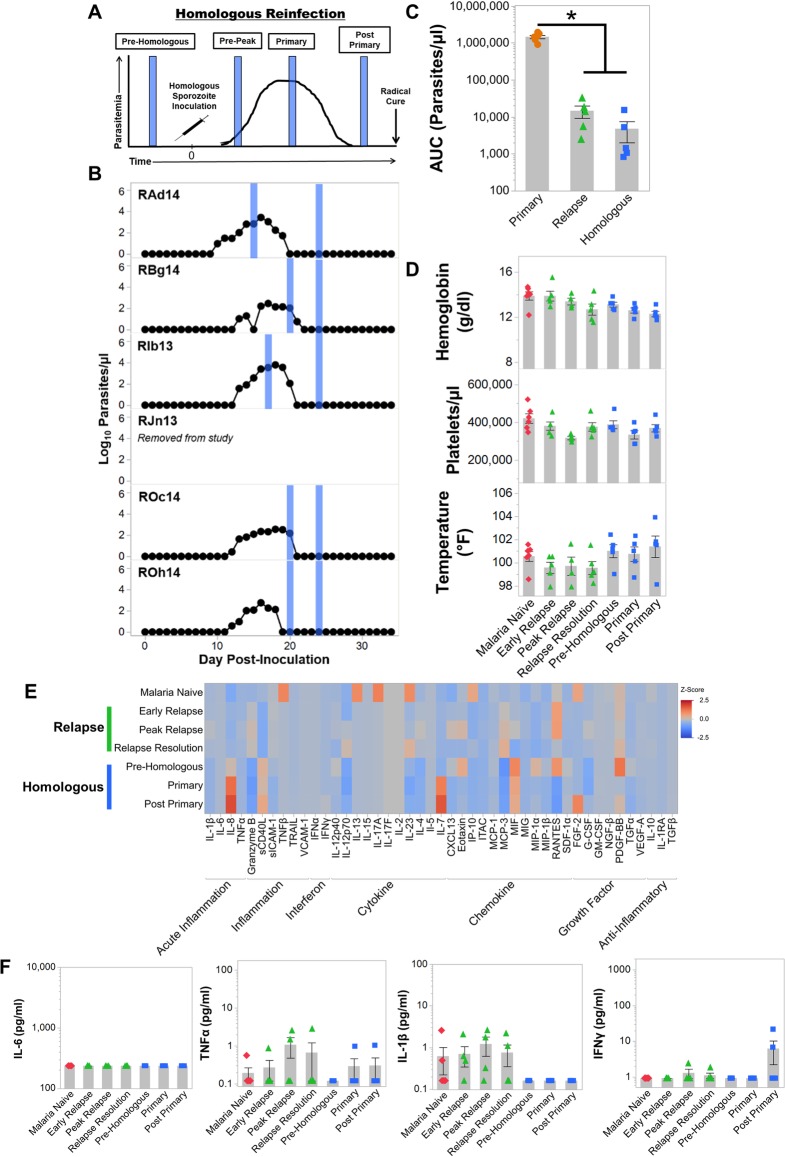
Reinfections with the same *P*. *cynomolgi* strain do not result in illness or inflammation. (a) Idealized study design for collecting specimens at different points during a homologous challenge of macaques previously infected with *P*. *cynomolgi* M/B strain. (b) Parasite kinetics over a 35-day *P*. *cynomolgi* M/B strain homologous challenge in rhesus macaques as determined by Giemsa-stained blood smears. Each row represents a different individual. Colored bars (blue) indicate when sample collections were performed. These collections correspond from left to right with the idealized experimental schematic in panel a. (c) Cumulative parasitemia as determined by area under the curve analysis. (d) Mean hemoglobin levels, reticulocyte concentrations, platelet concentrations, and temperature when animals were malaria naive (pink diamonds), during relapses (green triangles) and during homologous challenges (blue squares). Gray bars are the mean of the data points. (e) Heatmap of z-score transformed cytokine, chemokine, and growth factor concentrations at each infection point during relapses and the homologous challenge. Relapse concentrations are the same as shown in Fig 1. (f) Plasma concentrations of selected cytokines during relapses and homologous reinfections. Statistical significance was assessed by a linear mixed-effect model using a Tukey-Kramer HSD post-hoc analysis. Asterisks indicate p-value < 0.05 in comparison to the malaria naïve condition unless otherwise noted. Error Bars = SEM.

We questioned whether the animals would remain protected months later against a homologous parasite challenge infection and, if so, if the immune responses would be similar to those observed during a relapse. The same cohort of macaques was administered two rounds of radical cure to best ensure elimination of all liver- and blood-stage parasites. Approximately 60 days later they were re-challenged with 2,000 *P*. *cynomolgi* M/B strain sporozoites. Specimen collections were performed according to the schematic shown in Fig 5A and S4 Table. The reinfections with the homologous strain reached patency between days 9–12 post infection (mean ± SE = 11 ± 0.54 (Fig 5B). Peak parasitemias were substantially reduced compared to the initial infection and ranged from 269–5,742 parasites/μl (Fig 5B and 5C). There was no evidence that the decrease in parasitemia was directed against the sporozoite or liver stages since the number of days to patency was similar between the initial infections and homologous reinfections (S8 Fig). Like relapses, homologous reinfections did not cause clinical signs of malaria (Figs 5D and S9). Changes in cytokine profiles were also minimal with only IL-7 and RANTES differing significantly from pre-homologous values (Fig 5E). The pyrogenic cytokines IL-6, TNF-α, IL-1β, or IFN-γ did not increase during homologous reinfection and were not significantly different from values obtained during relapses (Fig 5F). Together, this homologous challenge experiment demonstrated that non-sterilizing immunity persisted for at least 60 days after radical cure and that this immunity could control peripheral parasitemia and the clinical manifestations of malaria in the *P*. *cynomolgi* model.

### Memory B cell responses suppress parasitemia and clinical disease during homologous reinfections

**Fig 6:**
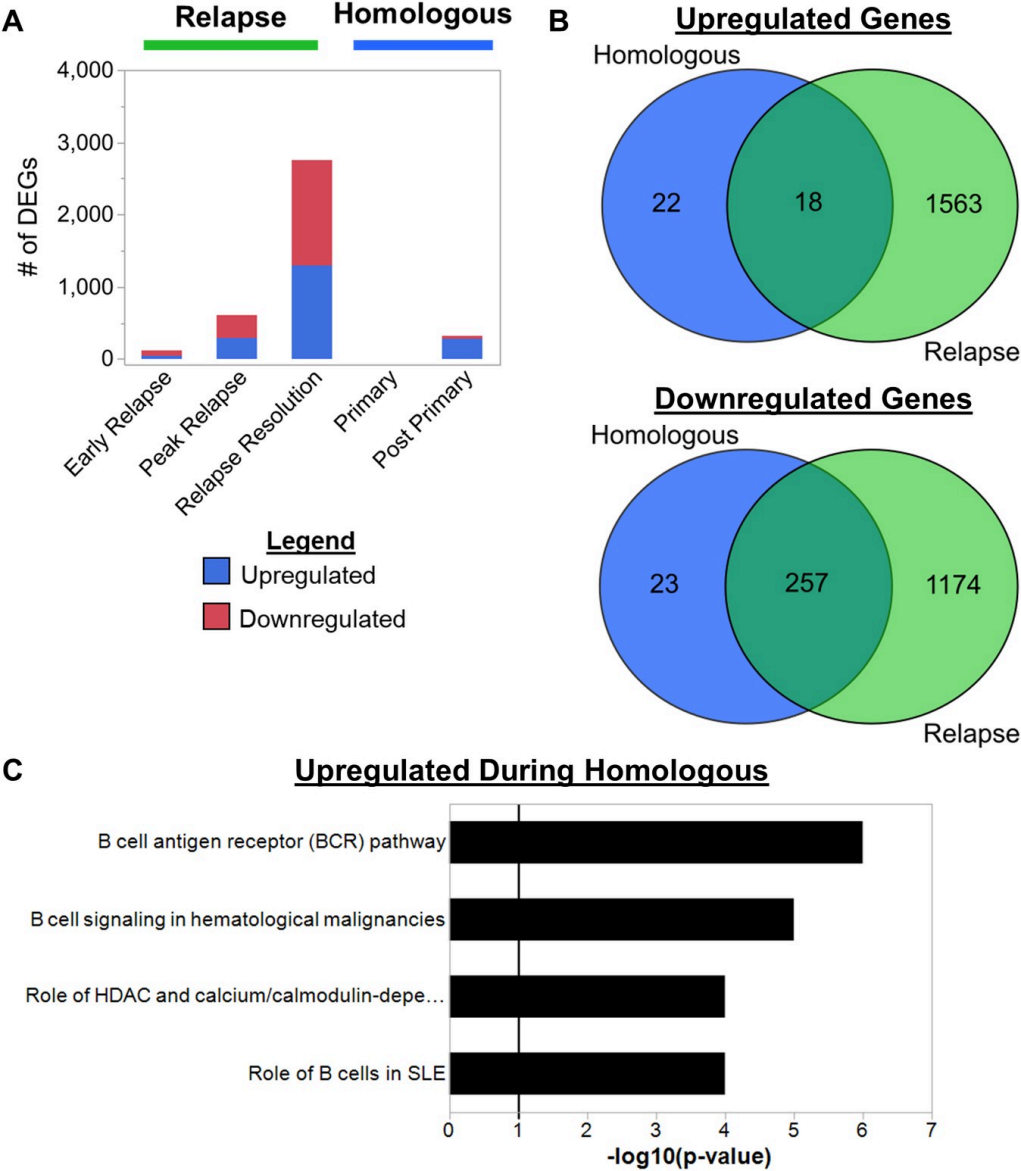
Homologous reinfections with *P*. *cynomolgi* cause changes in the host transcriptome related to B cells. (b) The number of differentially expressed genes identified at each infection point during relapses and homologous reinfections. (b) Venn diagrams showing the overlap of upregulated and downregulated differentially expressed genes during relapses and homologous reinfections. (c) The gene pathways identified using the upregulated differentially expressed genes during homologous reinfections. The solid black line in the pathway plots indicate an FDR corrected p-value of less than 0.05.

Since relapses and homologous reinfections had similar clinical presentations, we next employed RNA-Seq analysis on whole blood collected during the homologous challenges to determine if the host responses were similar. To identify DEGs during the homologous reinfections, the primary and post primary time points were compared to the pre-homologous challenge time point by ANOVA followed by a t-test post-hoc analysis with Benjamini-Hochberg false discovery rate (FDR) correction. Genes with an FDR adjusted p-value of less than 0.05 were considered significantly differentially expressed. As with the relapses, the homologous reinfections induced minimal changes in the host transcriptome (Fig 6A). Forty-five percent (18/40) of the upregulated DEGs during the homologous reinfections overlapped with the upregulated DEGs during relapses (Fig 6B). Pathway enrichment analysis of all upregulated DEGs during the homologous reinfections again identified pathways related to B cells (Fig 6C). The genes that were enriched in these pathways include those encoding B cell surface proteins such as CD19 and CD20 and B cell signaling molecules such as AKT, BTK, PLC-gamma2, and VAV-2 (S5 Table). Similar to relapses, these data indicate that the changes in the host responses during homologous reinfections were characterized by pathways involving B cells.

**Fig 7:**
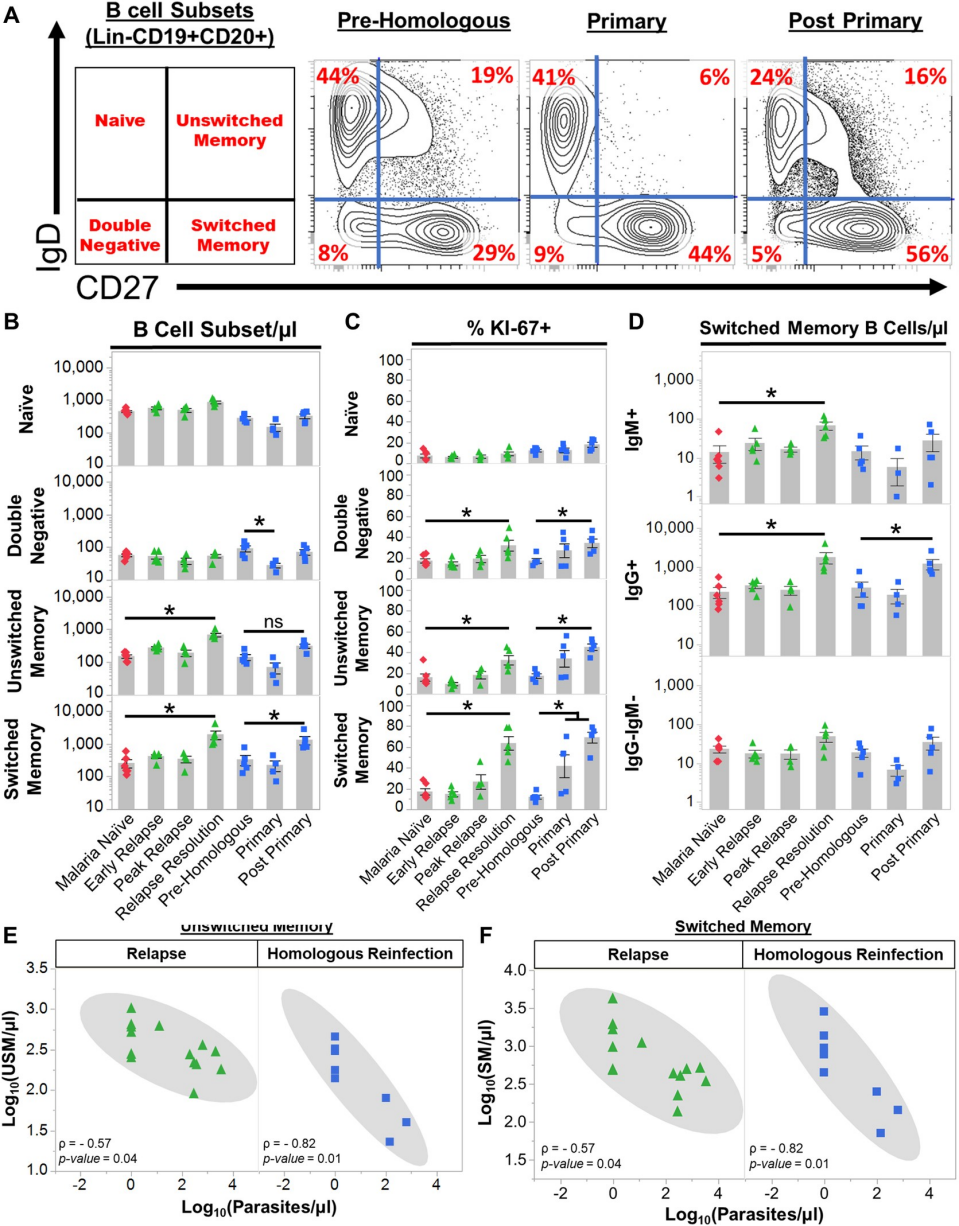
Homologous reinfections with *P*. *cynomolgi* induce an expansion of switched memory B cells. (a) The frequencies of four peripheral blood B cell subsets in rhesus macaques during homologous reinfections as determined by flow cytometry. A representative sample is shown. The far left panel indicates the B cell subset in each quadrant based on the gating strategy in S3 Fig. Red numbers in each quadrants indicate the percentage of each subset out of Lin-CD19+CD20+ B cells. (b) Absolute numbers of four B cell subsets at the indicated infection points. (c) The percentage of each B cell subset that is KI67+ at each infection point as determined by flow cytometry. (d) The absolute number of IgG+, IgM+, and IgG-IgM- switched memory B cells during relapses and homologous reinfections as determined by flow cytometry. (e) Spearman’s correlation analysis of the number of unswitched memory B cells and parasitemia during relapses and homologous reinfections. (f) Spearman’s correlation analysis of the number of switched memory B cells and parasitemia during relapses and homologous reinfections. Pink diamonds = malaria naïve, green triangles = relapse infections, blue squares = homologous reinfections. Gray bars indicate the mean of the data points shown; Error Bars = SEM. Statistical significance was assessed by a linear mixed effect model using a Tukey-Kramer HSD post-hoc analysis. Asterisks indicate a p-value < 0.05. ρ = Spearman’s correlation coefficient.

The changes in B cell subsets during the homologous challenge experiment were similar to those measured in relapse infections. There was an increase in SM B cells when the homologous infection was resolving at the post-primary time point (Fig 7A and 7B). However, unlike relapses, there was not a significant increase in USM B cells, and DN B cells significantly decreased during the homologous challenges (Fig 7B). The frequency of Ki67+ SM, USM, and DN B cells increased during the homologous reinfections like in the relapses although the USM and DN B cells did not increase in number (Fig 7C). Notably, only IgG+ SM B cells increased during the homologous reinfections whereas both IgG+ and IgM+ SM increased during relapses (Fig 7D). Importantly, USM, SM, and DN B cell numbers were inversely correlated with parasitemia during the homologous infections (Figs 7E, 7F and S10).

**Fig 8:**
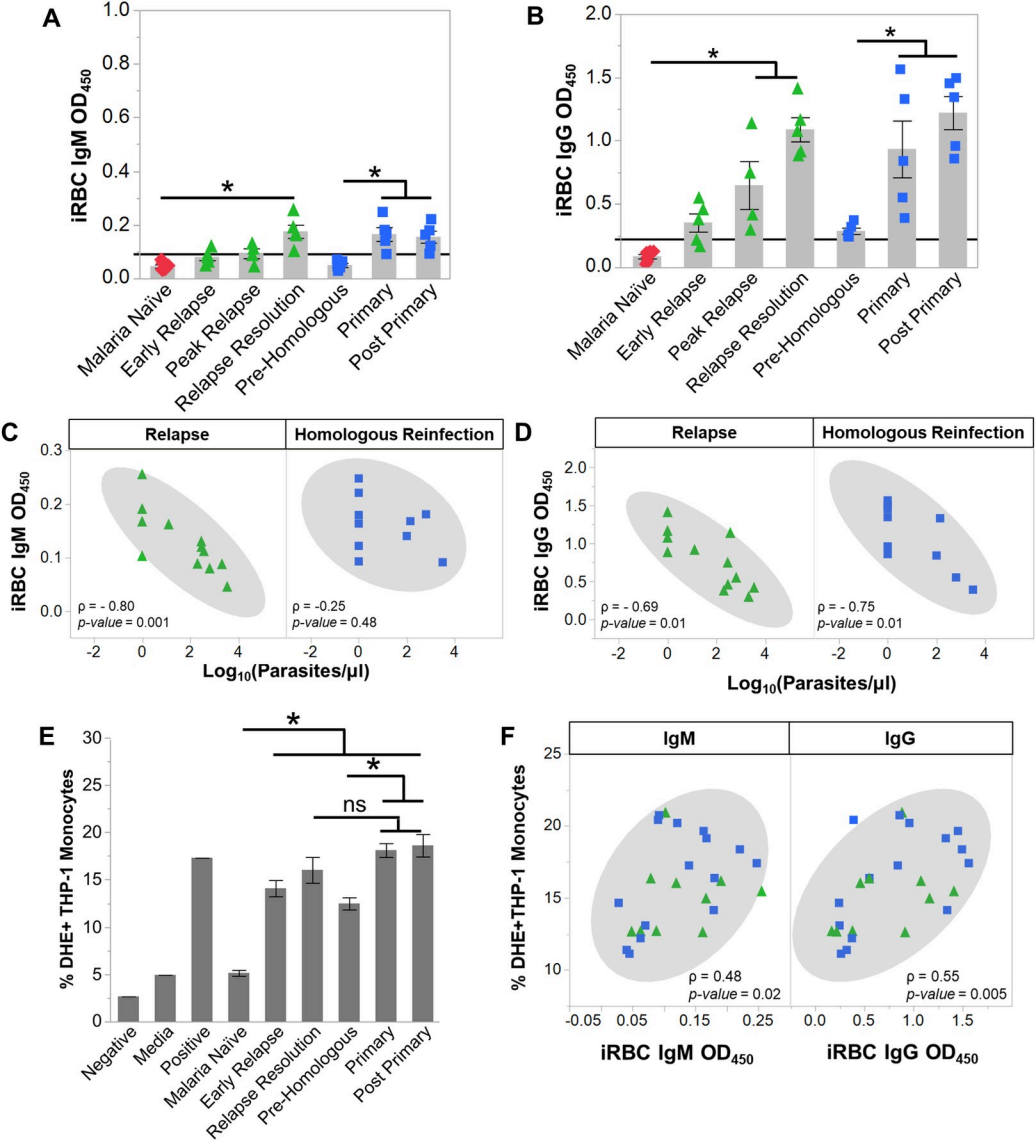
Anti-parasite IgG1 is produced during homologous reinfections and opsonizes *P*. *cynomolgi* infected RBCs as in relapses. Kinetics of anti-iRBC IgM (a) and anti-iRBC IgG (b) response during relapses and homologous reinfections as determined by ELISA; the black line indicates background defined as the average of the malaria naïve time point plus three standard deviations. Spearman’s correlation analysis of anti-iRBC IgM (c) and anti-iRBC IgG (d) responses in relation to parasitemia during relapses and homologous reinfections. (e) Percentage of THP-1 monocytes that phagocytosed *P*. *cynomolgi* M/B strain iRBCs after opsonization with heat-inactivated plasma collected at different infection points during relapses homologous reinfections. (f) Spearman's correlation analysis of opsonic phagocytosis activity and anti-iRBC IgM and IgG during relapses and homologous reinfections. Gray bars indicate the mean of the data points shown; Error Bars = SEM. Statistical significance was assessed by a linear mixed effect model using a Tukey-Kramer HSD post-hoc analysis. Asterisks indicate a p-value < 0.05. ρ = Spearman’s correlation coefficient.

Although total IgM was unchanged during the homologous reinfections, IgM recognizing both iRBCs and uRBCs was significantly increased as observed during relapses, albeit at much lower levels than the initial primary infections (Figs 8A, S11A and S12A). Consistent with relapses, total IgG and iRBC-specific IgG were also increased during homologous reinfections (Figs 8B and S11B). Again, the IgG subclass was predominantly IgG1 (S11C Fig). Similar to the relapses, iRBC-specific IgG was inversely correlated with parasitemia during the homologous reinfections, but iRBC-specific IgM was not (Fig 8C and 8D). Notably, the IgG reactivity with iRBC versus uRBC lysates was significantly higher in the homologous reinfections, like the relapses (S12B Fig).

As with the relapses, the humoral response during the homologous reinfections was highly effective at opsonizing iRBCs (Fig 8E). The increase in phagocytic activity was again correlated with iRBC-specific IgG (Spearman’s ρ = 0.55, p = 0.0005), but unlike relapses, iRBC-specific IgM was also correlated (Spearman’s ρ = 0.48 p = 0.02) with opsonic phagocytosis activity during homologous reinfections (Fig 8F). Altogether, these data are consistent with B cell mediated immune responses conferring protection during relapses and homologous reinfections.

### Clinically silent relapses harbor gametocytes

Next, we questioned how the immunity during a relapse may affect the number and proportion of asexual and sexual parasite stages during relapses and, thus, enumerated the sexual and asexual parasites by microscopy during the primary and relapse infections. During the primary infections, the parasite differentials were performed from patency until the peak of parasitemia. Samples after the peak were excluded from the analysis since these were collected after sub-curative blood-stage drug treatment. The parasites were enumerated during relapses for all days showing patent parasitemia.

**Fig 9:**
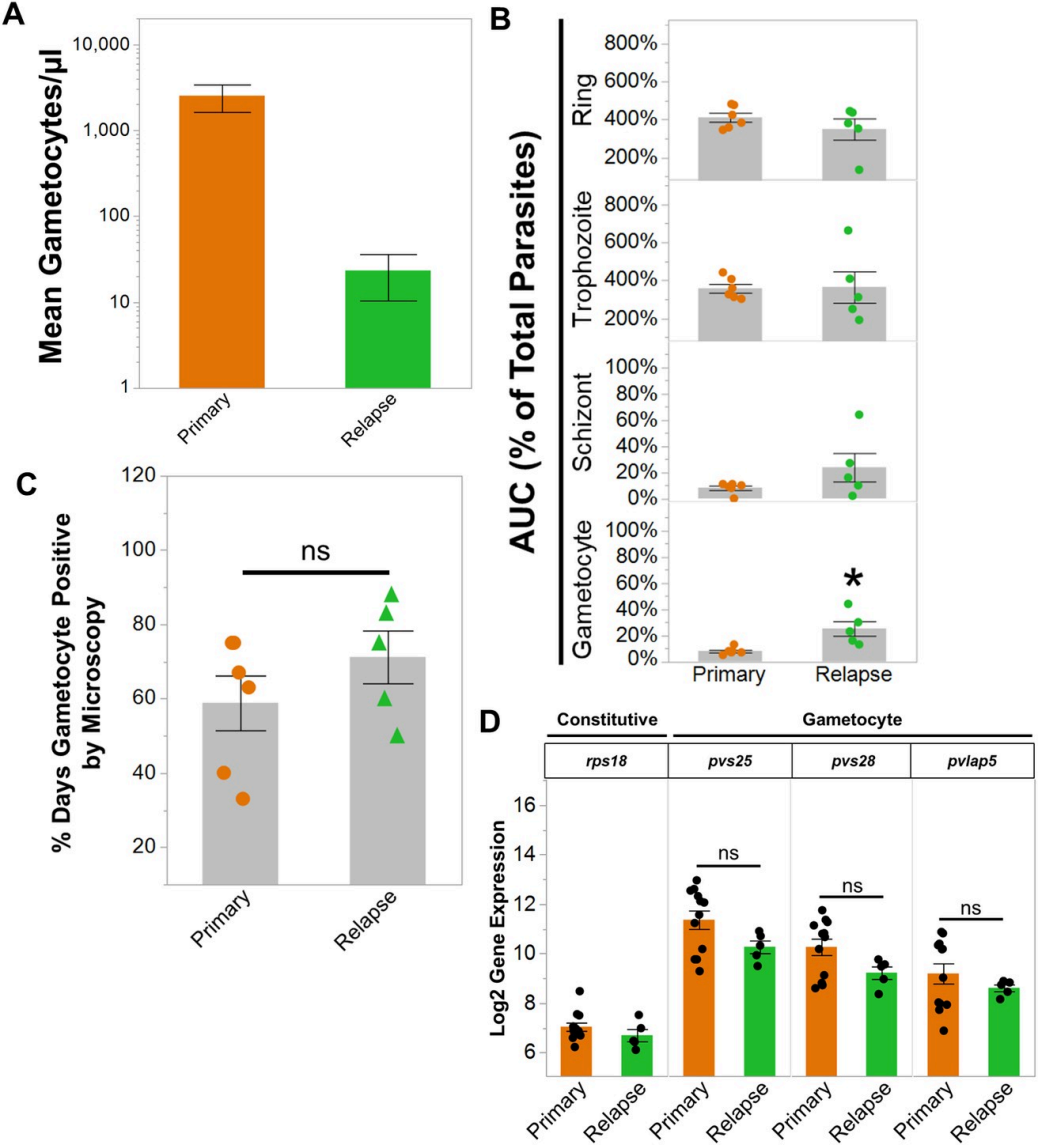
Clinically silent *P*. *cynomolgi* relapses harbor gametocytes. (a) Absolute number of gametocytes during primary and relapse infections as determined by microscopy. (b) The area under the curve of the proportions of rings, trophozoites, schizonts, and gametocytes during primary and relapse infections. The cumulative percent area exceeds 100% for rings and trophozoites because the infections were more than a single day. (c) The percentage of days out of the primary and relapse infections when gametocytes were observed by microscopy. (d) Gene expression of representative constitutive and gametocyte genes as determined by RNA-Seq during primary and relapse infections. All gametocyte genes are *P*. *cynomolgi* homologues of *P*. *vivax* genes implicated in transmission; the *P*. *vivax* gene annotation is listed for simplicity. Bars in all graphs indicate the mean log_2_ gene expression of the data points shown or average percent of each stage; Error Bars = SEM. Statistical significance was assessed by a paired t-test. Asterisks indicate a p-value < 0.05; ns = not significant.

As expected, the number of gametocytes were significantly reduced during the relapses given the significant reduction in parasitemia during relapses compared to the primary infections (Fig 9A). While the absolute number of gametocytes decreased, the cumulative proportion of circulating iRBCs that developed into gametocytes was significantly increased in the relapses (Fig 9B). In contrast, there was no significant difference between primary and relapse infections in the cumulative proportion of iRBCs containing ring, trophozoite, and schizont stages (Fig 9B). Notably, the percentage of days out of the primary and relapse infections that gametocytes were observed in the blood was also similar (Fig 9C). These data suggested that the immunity during relapses may disproportionately affect asexual stages as opposed to gametocytes.

To validate the microscopy results, we examined gametocyte gene expression using parasite transcriptomes obtained from whole blood RNA-Seq data. Samples with less than 100,000 parasite reads were removed from the analysis; these included some relapse samples. The post-peak time points from the primary infection were also excluded since these were collected after sub-curative antimalarial treatment. We limited the analysis to *P*. *cynomolgi* genes that are homologous to *P*. *vivax* gametocyte genes that have been associated with *P*. *vivax* transmission *in vivo* [38–40]. In concordance with the microscopy data, *P*. *cynomolgi* homologues of the mature gametocyte genes *pvs25*, *pvs28*, and *pvlap5* had similar gene expression across the primary infections and relapses (Fig 9D). Overall, these results demonstrate that despite the development of effective B cell immunity and reduction of parasitemia during relapses, the relapses maintained detectable levels of gametocytes.

## Discussion

In this study, single, sporozoite-initiated infections with *P*. *cynomolgi* in a cohort of rhesus monkeys resulted in the establishment of immunity that was capable of suppressing parasitemia during relapses or homologous reinfections initiated 60 days after radical cure. Whole blood RNA-Seq analysis showed that the host response during relapses and homologous reinfections was associated with distinct changes in the host transcriptome related to B cells. This finding was corroborated by flow cytometry and antibody ELISAs demonstrating that class-switched memory B cells rapidly responded during relapses and homologous reinfections along with a concomitant increase in anti-iRBC IgG. Collectively, these data demonstrate that protective, but non-sterilizing, humoral immunity can form after a single *P*. *cynomolgi* infection and may be key for preventing disease during relapses. Similarly, studies have found *P*. *vivax*-specific B cells can persist for years after an initial infection, and investigations using the *P*. *chabaudi* mouse model of malaria have also confirmed that humoral immunity can form and protect against subsequent challenges [41–44]. Therefore, we speculate that additional factors such as the genetic diversity of local *P*. *vivax* populations may be responsible for circumventing immunity, leading to symptomatic relapse infections. This seems likely when considering the high genetic diversity of *P*. *vivax* relapse infections in endemic areas [45–48]. Additional factors that may contribute to the lack of or subversion of immunity during a relapse include the age of first exposure or the presence of co-infecting pathogens.

Antibody responses during *Plasmodium* infection have been studied for decades, yet the roles of antibody isotypes other than IgG are currently under investigation [49, 50]. The inverse association of *P*. *cynomolgi*-specific IgM antibodies with relapse parasitemia indicate these antibodies may be involved in suppressing parasitemia and preventing the development of disease. USM B cells can differentiate to produce IgM in secondary responses, and these cells expanded during relapses and were inversely correlated with parasitemia during relapses and homologous reinfections [51, 52]. IgM+ SM B cells also expanded during relapses. Together, our data and recent evidence from rodent malaria models and human samples support a role for IgM+ memory B cells in anti-*Plasmodium* immunity [53]. Future studies should aim to evaluate neutralizing IgM responses to identify the origin of the B cell subsets that are responsible for their production. Such experiments are needed to delineate if anti-parasite IgM antibodies arise from the memory B cell compartment during recall responses or if they originate from naïve B cells that are stimulated to differentiate and secrete IgM during each blood-stage infection. Identification of the B cell compartment where protective antibodies originate and persist is needed to understand naturally acquired immunity against relapsing malaria parasites and may help to advance the development of a *P*. *vivax* vaccine.

While IgM may play a role in neutralizing blood-stage parasites during relapses, parasite-specific IgG1 was the predominant isotype produced during both relapses and homologous reinfections. Typically IgG3 rather than IgG1 has been reported as being the dominant subclass in human malaria [54, 55]. This discrepancy is likely due to differences between the NHP and human immune systems whereby IgG1 mediates the majority of effector functions in NHPs compared to humans where IgG1 and IgG3 contribute similarly [56]. In the data reported here, IgG levels strongly correlated with opsonic phagocytosis activity of *P*. *cynomolgi* iRBCs, an important mechanism of peripheral parasite control during blood stage infection. IgG+ SM B cells were the most significantly expanded memory subset during relapses and homologous reinfections and were inversely correlated with parasitemia in both cases, showing the potential importance of the SM B cell compartment during *P*. *cynomolgi* relapses and homologous reinfections. This should be taken into consideration with the ‘anti-relapse’ vaccine strategies currently being considered [57].

Despite the beneficial roles of IgG and IgM during *P*. *cynomolgi* infection, these antibodies may also contribute to pathogenesis. Removal of uRBCs by the immune system is a substantial contributor to the development of malarial anemia in humans and NHP models [36, 58–61]. Malarial anemia has been associated with the production of anti-self antibodies that tag uRBCs for elimination in rodent models and in human *P*. *falciparum* and *P*. *vivax* infections [62–64]. In this study, IgM and IgG antibodies that recognized uRBC lysates were detected during the primary infections, relapses, and homologous reinfections. The peak of the anti-uRBC IgM response occurred when parasitemia plateaued, and these antibodies were inversely correlated with hemoglobin levels when anemia was observed. Together, these data are consistent with a role for anti-uRBC IgM and IgG antibodies in the development of malarial anemia in addition to parasite control. Production of anti-uRBC antibodies could be due to non-specific, polyclonal activation of B cells in response to inflammatory stimuli released by iRBCs. Alternatively, the production of these antibodies may be an adverse, yet necessary, component of the normal immune response against *P*. *cynomolgi* that provides benefits like parasite neutralization. Either way future studies should identify the origin and function of ‘anti-self’ antibodies produced during longitudinal *Plasmodium* infections in NHPs.

Human studies with *P*. *vivax* have documented that gametocytes are present in symptomatic and asymptomatic relapse infections [7, 23, 24]. However, it has remained unclear how gametocytes are affected in the face of ongoing immune responses during a relapse [65]. Our study showed that *P*. *cynomolgi* gametocytes are substantially reduced during asymptomatic *P*. *cynomolgi* relapses, but the cumulative proportion of gametocytes increases relative to asexual stages of the parasite. The asexual stages predominated in circulation, and their relative proportions remained similar between the primary and relapse infections. These results argue that the reduction in the number of gametocytes is likely due to removal of asexual parasites, thereby, preventing their development into gametocytes, rather than anti-gametocyte immunity during relapses. In essence, our data are consistent with the host developing immunity to reduce parasitemia and prevent disease while the parasite manages to produce gametocytes that remain in circulation for ingestion by mosquitoes. This situation is advantageous for relapsing malaria parasites because the establishment of non-sterilizing immunity minimizes the chances of the host succumbing to infection while allowing for continued opportunities for transmission. Importantly, this scenario is fitting with the biology of *P*. *vivax* gametocytes since these are detectable soon after patency and, thus, could be transmitted before relapse parasitemia is substantially reduced [66]. Future studies should address whether the potent humoral immune response during *P*. *cynomolgi* relapses in rhesus macaques may also possess transmission-enhancing properties as shown previously in Toque monkeys (*Macaca sinica*) infected with *P*. *cynomolgi* [67].

While our study provides the most comprehensive analysis of *P*. *cynomolgi* relapses to date, it is not without limitation. Although our data strongly support the premise that humoral immunity is important in suppressing parasitemia during relapses to ameliorate disease, other cell types are likely involved. For example, future analysis of monocytes and T cells would be useful, particularly since the decreased inflammation observed during relapses and homologous reinfections could result in improved T cell help for B cells, thereby, increasing the effectiveness of the humoral immune response in subsequent exposures. Nonetheless, our study demonstrates the importance of humoral immunity because the increase in IgG during relapses and homologous reinfections occurs nearly twice as fast as in primary infections. Also, the lower increase in the IgM response during relapses and reinfections compared to the primary infections is consistent with a strong secondary response. Second, this study was not designed to test the infectiousness of gametocytes to mosquitoes. However, this would be an important addition to *P*. *cynomolgi* relapse investigations based on our results. Lastly, although *P*. *cynomolgi* infections of rhesus macaques are a valuable experimental surrogate for human *P*. *vivax* infections, there are differences that may influence the development of immunity. For example, *P*. *cynomolgi* parasitemias in rhesus are typically higher than *P*. *vivax* parasitemias in humans, which could lead to establishment of durable immunity faster in the rhesus macaque—*P*. *cynomolgi* infection model. On the other hand, experimental infections with *P*. *vivax* in neurosyphalitic patients have demonstrated appreciable homologous immunity months to years after one infection [68–70]. The results from those studies argue that the data presented here have a high degree of relevance to *P*. *vivax* infections in humans.

In conclusion, our studies with *P*. *cynomolgi* in rhesus macaques and studies on human *P*. *vivax* infections collectively provide strong evidence that relapses and homologous reinfections do not necessarily result in clinically detectable disease [41, 68–70]. Instead, it is becoming clear that relapses and potential reinfections with the same parasite variant can be clinically silent, and we have shown that this is, at least in part, due to potent humoral immunity that forms after an initial infection. This is highly significant considering that we have shown that clinically silent *P*. *cynomolgi* relapses continue to harbor gametocytes. If individuals in endemic communities have clinically silent relapses, they will not seek treatment. Meanwhile, they may serve as a source of gametocytes that may remain infectious to mosquitoes. The number of clinically silent relapse infections and their infectiousness to mosquitoes remains largely unknown and should be evaluated carefully in the future. As a next step on the path to eliminating *P*. *vivax* and other relapsing malaria parasites, empirical studies that identify the factors that influence relapse pathogenesis, immunity, and infectiousness to mosquitoes are needed, and the *P*. *cynomolgi*-macaque models can be used for investigations in each of these areas.

## Materials and methods

### Animal use

Nonhuman primate cohort infections were performed at the Yerkes National Primate Research Center (YNPRC) at Emory University, an Association for Assessment and Accreditation of Laboratory Animal Care (AAALAC) international-certified institution. Freshly isolated, salivary gland sporozoites were generated for each infection using additional rhesus monkeys at the Centers for Disease Control and Prevention (CDC). Rhesus monkeys utilized for experiments were of Indian origin, male, 7–13 kg, and 5–6 years of age. All male animals were used for experiments to eliminate the female menstrual cycle as a contributor to the development of anemia. All procedures including blood collections, infections with malaria parasites, clinical interventions, etc. were reviewed and approved by Emory University’s and/or CDC’s Institutional Animal Care and Use Committees. During the experimental procedures at YNPRC, the animals were housed socially in pairs in compliance with Animal Welfare Act regulations as well as the Guide for the Care and Use of Laboratory Animals.

### Parasite isolates

*Plasmodium cynomolgi* M/B strain parasites were used for all experiments as previously described [12].

### Sporozoite generation and inoculation

Two thousand freshly isolated, salivary gland sporozoites were generated, isolated, and administered intravenously as previously described to initiate the initial infections with relapses and homologous reinfections [12].

### Drug treatment regimens

Subcurative antimalarial treatments consisted of a single dose of artemether at 1 mg/kg administered intramuscularly (IM). Curative blood-stage treatments consisted of a 7 day regimen of artemether administered IM with the first dose at 4 mg/kg and subsequent doses at 2 mg/kg. Radical cure consisted of a combination treatment with artemether and primaquine. Artemether was first administered IM for 8 days using an initial dose of 4 mg/kg followed by subsequent doses at 2 mg/kg. At the conclusion of the artemether treatment, primaquine was administered orally in peanut butter at 2 mg/kg for 7 days.

### Sample collections

Blood was collected in EDTA at pre-defined time points as indicated in the experimental schematics in Figs 1A and 5A. Parasitemia and hematological parameters were evaluated daily by light microscopy and complete blood counts (CBC) analysis, respectively. For the daily parasitemia and CBC assays, blood was collected into a pediatric capillary tube using a standardized ear-prick procedure as previously described [12]. Blood specimens utilized for transcriptomic analysis were bled directly into Tempus tubes according to the manufacturer’s suggested protocol. Plasma was collected from each time point prior to isolation of PBMCs for flow cytometry analysis as described below. Bone marrow samples were not utilized for the experiments presented here.

### Parasite enumeration

Daily parasitemia was determined as reported in Joyner et al. 2016 [12]. Briefly, thick and thin blood film preparations from capillary or venous blood were prepared and allowed to dry. Thin films were fixed with 100% methanol, and thick films left unfixed. The thick and thin films were then stained using a Wright’s-Gurr stain. For thick film preparations, parasites were enumerated by determining the number of parasites that were observed within 500–2000 white blood cells (WBCs) depending on the parasite density. The number of parasites per the number of WBCs was then calculated and multiplied with the leukocyte count as determined by the CBC to yield parasites per microliter. For days where parasitemia was too high to enumerate using thick blood films, the thin blood film was used; this was typically when parasitemia was greater than 1%. The number of parasites out of 1000–2000 RBCs were determined and the percent parasitemia calculated by dividing the number of parasites counted by the number of total RBCs counted and multiplying by 100. The percentage of infected RBCs was then multiplied against the RBC concentration as determined by the CBC analysis to determine parasites/μl. Parasitemia was determined by two expert microscopists independently through the course of each infection. If discrepancies were observed between the two readers, a third, independent microscopist counted the slides. The two most similar values were then averaged to determine the parasitemia at any point during infection.

### Parasite differentials

The parasite stages present at the selected times during the infections were determined by thick or thin film microscopy by counting 10 to 100 parasites and noting their stage. Each slide was examined for at least 15 minutes before stopping. The proportion of rings, trophozoites, schizonts, and gametocytes, were then calculated and used to determine the frequency of each parasite-stage per microliter of blood using the number of parasites per microliter as determined above or as proportions (%) for area under the curve (AUC) analysis as described below.

### Area under the curve

Area under the curve (AUC) for parasitemia (parasites/μl) and proportions (%) of parasite stages during the primary, relapse, and/or homologous reinfections were calculated using Riemann sums by determining the trapezoidal area between each data point during the indicated time periods. The formula used to calculate AUC was as follows: $\left(\frac{\mathrm{Data}\;\mathrm{Point}\;1+\mathrm{Data}\;\mathrm{Point}\;2}{2}\right)\times \Delta \mathrm{time}$.

### Complete blood count and temperature data collection

Complete blood counts (CBCs) were performed prior to infections and daily after inoculation using capillary and/or venous blood. If values from the CBC were considered abnormal (e.g. low platelet counts or observation of nucleated RBCs), the values obtained by the hematology analyzer were either confirmed or adjusted based on a manual differential or manual platelet count. For manual differentials, the phenotype (e.g. monocyte, neutrophil, nucleated RBC, etc.) was determined, and the percentage of each subset calculated. If there was a discrepancy with the CBC based on the differential, the percentage of monocytes, lymphocytes, and granulocytes was adjusted to ensure accuracy. If nucleated RBCs were present, the number of nucleated RBCs was determined and subtracted from the leukocyte count and added to the RBC count. Rectal temperatures were also obtained when animals were sedated for sample collections. Notably, two pre-infection values were collected prior to the initial infections to ensure accurate naive measurements were obtained since abnormal values may occur before an NHP becomes used to daily interaction. These values were averaged to obtain the malaria naïve values used for the analysis of the clinical data.

### Multiplex cytokine arrays

A custom, nonhuman primate multiplex cytokine assay was designed and purchased from eBioscience/Affymetrix, which is now a part of Thermofisher. These kits were performed according to the manufacturer’s suggested protocol except for one modification. Instead of diluting plasma 1:1 with sample dilution buffer, the samples were not diluted prior to running the assay. This was altered after initial experiments demonstrated that many analytes were not within the dynamic range of the standard curves if additional dilutions were performed. Samples were fully randomized prior to performing the multiplex kit to minimize plate- and well-specific effects. All multiplex data was analyzed using the ProcartaPlex Analyst software available through Thermofisher. Concentrations of cytokines in the plasma were determined and used for downstream analyses.

### RNA-Sequencing library preparation and sequencing

Total RNA was extracted using the Tempus RNA isolation kit (Fisher Scientific; Cat#:4380204). Globin transcripts were depleted using GLOBINclear Human Kit (Fisher Scientific; Cat#:AM1980) according to the manufacturer's instructions. Libraries were prepared using the Illumina TruSeq mRNA stranded kit (Illumina Inc.; Cat#:20020595) as per manufacturer’s instructions. 1 ug of Globin depleted RNA was used for library preparation. ERCC (Invitrogen; Cat#:4456740) synthetic spike-in 1 or 2 was added to each Globin depleted RNA sample. The TruSeq method (high-throughput protocol) employs two rounds of poly-A based mRNA enrichment using oligo-dT magnetic beads followed by mRNA fragmentation (120–200 bp) using cations at high temperature. First and second strand cDNA synthesis was performed followed by end repair of the blunt cDNA ends. One single “A” base was added at the 3’ end of the cDNA followed by ligation of barcoded adapter unique to each sample. The adapter-ligated libraries were then enriched using PCR amplification. The amplified library was validated using a DNA tape on the Agilent 4200 TapeStation and quantified using fluorescence based method. The libraries were normalized and pooled and clustered on the HiSeq3000/4000 Paired-end (PE) flowcell on the Illumina cBot. The clustered PE flowcell was then sequenced on the Illumina HiSeq3000 system in a PE 101 cycle format. Each sample was sequenced to a target depth of 100 million pairs (50 million unique fragments) with exception of Time point 2 samples that were sequenced to 200 million pairs (100 million unique fragments).

### RNA-Sequencing alignment

Raw FASTQ files from the RNA-Seq experiments of all animals at all time points were aligned to the *P*. *cynomolgi* [21] and *M*. *mulatta* (version 7.8) [71] reference genomes using the Spliced Transcripts Alignment to a Reference tool (STAR, version 2.4.1c). The aligned features were further quantified and mapped using the High-Throughput Sequencing tool version 0.6.1p1[72] using only the *P*. *cynomolgi* reference to select parasite-specific transcripts. All sequencing and transcript mapping results were deposited to the NCBI GEO and SRA databases under the accessions GSE104223 (E23) and GSE104101 (E24).

### RNA-Seq analysis of host transcripts

Raw count data from MaHPIC Experiments 23, 24, and 25 were all library size normalized using the ‘DESeq2’ package for R [73]. Prior to normalization for library size, genes of extremely low read count (<10 reads across all samples) were filtered. RNA-Sequencing data taken during initial infections, homologous reinfections, and heterologous reinfections (not presented here) were normalized together. Data structure was then examined with principal component analysis. Individual animal effects were removed using Supervised Normalization of Microarrays with the ‘SNM’ package for R [32]. LIMMA was then used to assess gene expression changes during each infection and between infection stages [74]. Fraction of gene expression variance explained by unsupervised Ward’s hierarchical cluster analysis was determined by finding the ratio of between-cluster variance (B) to total variance (T), which is in turn the sum of between-cluster and within-cluster variance (W).

$$B=\sum_{j}n_{j}\sum_{i}{\left(\bar{g_{j}}(i)-\bar{g}(i)\right)}^{2}$$

$$W=\sum_{k}\sum_{i}{\left(g_{k}(i)-\bar{g_{j}}(i)\right)}^{2}$$

T=B+W

$$VE=\frac{B}{T}$$

Where j = 1, 2, … are the different clusters, i = 1, 2, … are the different genes measured in each sample, k = 1, 2, … are the different samples, n_j_ is the number of samples in cluster j, $\bar{g_{j}}(i)$ is the average value of gene i in cluster j, $\bar{g}(i)$ is the average value of gene i across all samples, and *g_k_*(*i*) is the value of gene i in sample k. For the within-cluster variance equation, the cluster j that is used for each step of the summation is the cluster to which sample k belongs.

### RNA-Seq analysis of parasite gene expression

Only samples in which parasites were detected by microscopy and at least 100,000 total reads (corresponding to at least 90 parasite/ μL) were analyzed. For the comparison of initial infections versus relapses, the pre-peak and peak infection stages were used for comparison with the relapse time points. The early relapse or peak relapse points were used to represent relapses. For animals in which both early and peak relapse samples had sufficient parasitemia and parasite reads the earlier sample was selected to represent relapse. Notably, if there was not an early relapse infection stage, the peak relapse point was used for analysis. ROh14 did not have a relapse and thus was not analyzed. All samples were library size normalized together and log_2_-transformed using DESeq2. Changes in gene expression were then assessed by using a linear mixed effect model with a Tukey-Kramer HSD post hoc analysis. P-values < 0.05 were considered statistically significant.

### PBMC isolation

Plasma was isolated prior to performing the PBMC isolation by centrifuging the blood samples at 400 × g followed by pipetting off the plasma. After removing the plasma, the blood pellet was resuspended in two times the original volume of blood that was received. This modification of the procedure did not appear to alter the viability or yield of PBMCs. After this step, the manufacturer’s suggested protocol was followed. After each isolation, each monkey’s PBMCs were washed two times in sterile PBS followed by enumeration on a Countess II fluorescent cell counter. The viability of the PBMCs was simultaneously assessed by Trypan Blue exclusion assay. PBMC viability was always ≥ 90%.

### Flow cytometry

5×105–2×10^6^ PBMCs were aliquoted into flow cytometry tubes for staining with fluorescently conjugated antibodies. The variation in number of PBMCs used for each staining procedure was due to leukopenia that developed during the acute, symptomatic infections. After aliquoting into individual FACS tubes, cells were washed once more in PBS prior to re-suspending in antibody cocktails comprised of the antibodies indicated in S6 Table for the initial infections with relapses and S7 Table for the homologous reinfections. Notably, some markers listed are not presented in the manuscript, but are provided to convey the complete panel configurations used in each experiment.

All staining procedures were two-step. For surface IgG staining, the IgG was prepared in a separate cocktail and added first, followed by a 30-minute incubation, washing PBS by centrifugation at 400 × g, and then resuspending in a cocktail that contained the other antibodies in the panels.

For intracellular staining, cells were initially surface-stained with the cocktail followed by incubation in eBioscience FoxP3 fix perm buffer (Thermofisher) overnight at 4°C for intracellular markers. After fixing overnight, the cells were washed according to the manufacturer’s procedure and then incubated for 45 minutes at 4°C with antibodies against intracellular markers. The cells were then washed twice in the fix/perm buffer provided by the kit and resuspended in 100–200 μl of PBS depending upon cell yield. All samples were acquired on an LSR-II flow cytometer using standardized acquisition templates and rainbow calibration particles for voltage control. Compensation controls were run at each acquisition. Data were initially compensated in FlowJo version 10.1 followed by exporting to Cytobank for gating. Cell population level statistics were then exported from Cytobank for further analysis.

### Determining absolute cell counts with flow cytometry data

Absolute numbers for each B cell subset was determined by calculating the percentage of each subset out of the mononuclear cells in the sample and multiplying the percentage with the mononuclear cells/μl value obtained from the CBC at each time point. The mononuclear cells/μl was obtained by adding the lymphocyte/μl value to the monocyte/μl value. If two values for absolute numbers were obtained due to a population being present in both panels (e.g. switched memory), the values were treated as technical duplicates and averaged to obtain the final value used for each analysis.

### Total IgG and IgM Antibody ELISAs

Corning high-binding microtiter plates were coated with Anti-Monkey IgG+IgA+IgM (Rockland Immunochemicals) or Anti-Monkey IgM (Life Diagnostics) diluted in ELISA coating buffer (Abcam) to 0.6 ug/ml and 5 ug/ml, respectively. The plate was incubated overnight at 4°C followed by washing four times with PBS containing 0.05% Tween-20 (PBS-T). After the final wash, the plate was blotted dry and blocked using serum-free Sea Block (Abcam) for two hours at RT followed by four washes in PBS-T. Plasma samples from the different infection points were diluted 1:100,000 for total IgG or 1:10,000 for total IgM in 10–33% serum-free Sea Block and then added to each well. The plate was then incubated at RT for 2 h followed by washing four times with PBS-T. After blotting dry, HRP-conjugated anti-IgG (Jackson Immunoresearch) or HRP-conjugated anti-IgM (Jackson Immunoresearch) diluted 1:30,000 or 1:20,000 in 10–33% Sea Block in PBS, respectively, were added to each well and incubated for 1 h at RT in the dark. After incubating, the plate was washed four times with PBS-T and 100 μl of High Sensitivity TMB Substrate (Abcam) was added to each well and allowed to develop for 3–5 minutes. One hundred microliters of Stop Solution (Abcam) was added to stop the reaction. The absorbance at 450 nm was then measured, and total IgM or total IgG antibody concentrations were calculated based on a 4-PL standard curve using purified IgM calibrators from Abcam’s Monkey Total IgM ELISA kit and using purified IgG Monkey Calibrators from Rockland Immunochemicals. Concentrations of total IgG and IgM were used for downstream analyses.

### *In vitro* maturation and isolation of *P*. *cynomolgi* schizonts

Rhesus macaques were inoculated with cryopreserved, blood-stage *P*. *cynomolgi* B/M strain parasites to generate schizonts for lysate preps described below. Briefly, a vial of cryopreserved, blood-stage parasites were removed from the liquid nitrogen and quickly thawed in a 37°C water bath. After thawing, saline solutions of different concentrations were added drop-wise to slowly change the osmotic pressure while preventing RBC lysis. The number of ring-stage parasites were then enumerated using light microscopy as described above and inoculated intravenously into a rhesus macaque. The infections were followed daily for each monkey until parasitemia reached 3–10% ring-stage parasites. At this time, blood containing predominantly rings was collected in sodium heparin, washed, and depleted of leukocytes and platelets by passing over a glass bead column and through a Plasmodipur filter. The parasites were then matured *ex vivo* to 3–8 nucleated schizonts under blood-gas conditions (5%:5%:90%;O2:CO2:N2) in RPMI supplemented with L-glutamine, supplemented with 0.25% sodium bicarbonate, 50 μg/ml hypoxanthine, 7.2 mg/ml HEPES, 2 mg/ml glucose, and 10–20% Human AB+ serum. When mature, the schizonts were isolated by a 1.093 g/ml Percoll density gradient. The parasite layer was then isolated and washed 4 times in sterile RPMI, aliquoted, and stored in vapor phase liquid nitrogen until needed.

### Infected and uninfected RBC lysate preparation

Aliquots of parasite or uninfected RBC pellets were removed from liquid nitrogen storage, thawed quickly in a 37°C water bath and placed back into the liquid nitrogen tank for ten minutes. This procedure was repeated three more times. After the final thaw, 1 volume of PBS was added followed by vigorous vortexing for 1–2 minutes. The aliquot was then centrifuged at 3,000 × g for 10 minutes at 4°C. The supernatant was then removed and placed into another sterile tube and the pellet discarded. This process was repeated three more times. After the final centrifugation, the protein concentration was determined using a Pierce BCA assay according to the manufacturer’s protocols. The lysates were then diluted to optimal concentrations for ELISAs in PBS, aliquoted, and stored at -80°C until needed.

### iRBC- and uRBC-specific antibody ELISAs

Corning high-binding microtiter plates were coated with schizont lysate or uninfected RBC lysate diluted in ELISA coating buffer (Abcam) to 5 ug/ml. The plate was incubated overnight at 4°C followed by washing four times with PBS containing 0.05% Tween-20 (PBS-T). After the final wash, the plate was blotted dry and blocked using serum-free Sea Block (Abcam) for two hours at RT followed by four washes in PBS-T. Plasma samples from the different infection points were diluted 1:100 in 10–33% serum-free Sea Block and then added to each well. The plate was then incubated at RT for 2 h followed by washing four times with PBS-T. After blotting dry, horseradish-peroxidase (HRP) conjugated anti-IgG (Jackson Immunoresearch) or HRP-conjugated anti-IgM (Jackson Immunoresearch) diluted 1:30,000 or 1:20,000 in 10–33% Sea Block in PBS, respectively, were added to each well and incubated for 1 h at RT in the dark. After incubating, the plate was washed four times with PBS-T and 100 μl of High Sensitivity TMB Substrate (Abcam) was added to each well and allowed to develop for 3–5 minutes. One hundred microliters of Stop Solution (Abcam) was then added to stop the reaction. The absorbance at 450 nm was then measured, and the OD_450_ of iRBC and uRBC-specific IgG and IgM were used for downstream analyses.

### Parasite-specific IgG subclass antibody ELISAs

Corning high-binding microtiter plates were coated with schizont lysate diluted in ELISA coating buffer (Abcam) to 5 ug/ml. As a positive control, duplicate wells were coated with recombinant expressed rhesus IgG1, IgG2, or IgG3 (NHP Reagent Resource) diluted to 1 ug/ml in ELISA coating buffer. The plate was incubated overnight at 4°C followed by washing four times with PBS containing 0.05% Tween-20 (PBS-T). After the final wash, the plate was blotted dry and blocked using serum-free Sea Block (Abcam) for two hours at RT followed by four washes in PBS-T. Plasma samples from the different infection points were diluted 1:100 in 10% serum-free Sea Block and then added to each well. The plate was then incubated at RT for 2 h followed by washing four times with PBS-T. After blotting dry, mouse anti-rhesus IgG1, IgG2, or IgG3 (NHP Reagent Resource) diluted 1:10,000, 1:1,000, and 1:10,000 in 10% Sea Block in PBS, respectively, was added to each well and incubated at RT for 1 h. Following four washes in PBS-T and blotting dry, HRP-conjugated anti-mouse IgG (Jackson Immunoresearch) diluted 1:10,000 in 10% Sea Block in PBS was added to each well and incubated for 1 h at RT in the dark. After incubating, the plate was washed four times with PBS-T and 100 μl of High Sensitivity TMB Substrate (Abcam) was added to each well and allowed to develop for 30 minutes. One hundred microliters of Stop Solution (Abcam) was then added to stop the reaction. The absorbance at 450 nm was then measured, and optical densities (ODs) were used for downstream analyses.

### iRBC phagocytosis assay

We adapted a previously established phagocytosis assay for *P*. *cynomolgi* [75]. Briefly, the THP-1 monocytic cell line was obtained from ATCC and maintained in vented 75cm^2^ culture flasks at 10% CO2 in RPMI-1640 supplemented with 10% fetal bovine serum, 2mM L-glutamine, 10mM HEPES, 1mM sodium pyruvate, 4500 mg/L glucose, and 1500 mg/L sodium bicarbonate. The cells were maintained at a density of 1 × 10^5^ cells/ml of culture and were not allowed to exceed 1 × 10^6^ cells/ml. *Plasmodium cynomolgi* strain M/B were thawed, matured *in vitro* to schizonts, and isolated as described above. Purified schizonts were incubated with 5 ug/ml dihydroethidium (DHE) for 20 min at 37°C, followed by 3 washes in THP-1 media before use in the assay. After labeling with DHE, the schizonts were opsonized in heat-inactivated plasma from different specimen collections for 45 minutes at RT in the dark. While the parasites were opsonizing, THP-1 cells were harvested and an aliquot of THP-1 cells was incubated with 5 uM Cytochalasin D for 1 h at 37°C to serve as a negative control. THP-1 cells were then added to each well to a final Effector Target ratio of 1:20 and incubated at 37°C for 3 h. The cells were then transferred to FACS tubes, washed twice with THP-1 media, and then lysed with ACK lysing solution for 10 min at RT in the dark. Cells were then re-suspended in PBS and acquired immediately on a BD LSR-II using a standardized acquisition template.

### Statistical analyses

All statistical analyses were performed using a linear mixed-effect model with Tukey-Kramer HSD post-hoc analysis. For the statistical model, each animal was treated as a random effect with time points as fixed effects. All data were transformed as necessary to ensure the best model fit, and the best model fits were typically obtained with a log_10_, log_2_, or arcsin transformation.

### Data management and release

All data went through rigorous validation protocols and are publicly deposited in public repositories. All clinical data associated with the experiment have been publicly released on PlasmoDB http://plasmodb.org/plasmo/mahpic.jsp (see http://plasmodb.org/common/downloads/MaHPIC/Experiment_23/ and http://plasmodb.org/common/downloads/MaHPIC/Experiment_24/ for the datasets in this manuscript). All RNASeq results have been publicly released as described in the Materials and Methods. Flow cytometry, multiplex cytokine assays, and ELISA are publicly available at ImmPort as part of study SDY1409.

## Supporting information

S1 Fig Hemoglobin and platelet kinetics with parasitemia during initial and relapsing *P. cynomolgi* infections. Daily hemoglobin levels (a) and platelet numbers (b) during initial and relapsing *P*. *cynomolgi* M/B strain infections. The five-letter code on the left-hand side of each graph indicates a different individual rhesus macaques. k = multiply number shown by 1,000.
(TIF)

S2 Fig Concentrations of 45 cytokines, chemokine, and growth factors during *P. cynomolgi* primary infections, relapses and homologous reinfections. Statistical significance was assessed by a linear mixed effect model using a Tukey-Kramer HSD post-hoc analysis. Asterisks indicate a *p*-value < 0.05.
(DOCX)

S3 Fig Gating strategy and IgG/IgM surface profiles of four B cell subsets in the peripheral blood of rhesus macaques. (a) A representative gating strategy for monitoring rhesus macaque B cell subsets in PBMCs is shown. (b) Histograms of surface IgM and IgG expression of the four B cell subsets being monitored from the representative sample shown in panel a. (c) The average percentage of each B cell subset that are IgG+, IgM+, or IgM-IgG-USM from six malaria naïve rhesus macaques. USM = Unswitched Memory, DN = Double-Negative, SM = Switched Memory.
(TIF)

S4 Fig Granulocyte, monocyte and lymphocyte kinetics during initial and relapsing *P. cynomolgi* infections. The absolute number of granulocytes, monocytes, and lymphocytes per microliter of blood during initial infections and relapses as determined by complete blood counts. Pink diamonds = malaria naïve, orange circles = initial infection, and green triangles = relapse infection. Gray bars indicate the mean of the data points shown; Error Bars = SEM. Statistical significance was assessed by a linear mixed effect model using a Tukey-Kramer HSD post-hoc analysis. Asterisks indicate a *p*-value < 0.05.
(TIF)

S5 Fig Spearman’s correlation analysis of the relationship between naïve and double negative B cells during primary and relapse infections. ρ = Spearman’s correlation coefficient.
(TIF)

S6 Fig Total IgM, total IgG, and IgG subclass kinetics during primary and relapse *P. cynomolgi* infections. Kinetics of total IgM (a) and IgG (b) at different infection stages during initial infections and relapses as determined by ELISA. (c) IgG subclasses recognizing iRBC lysates as determined by ELISAs. The black line indicates background. Pink diamonds = malaria naïve, orange circles = initial infections, and green triangles = relapse infections. Bars indicate the mean of the data points shown; Error Bars = SEM. Statistical significance was assessed by a linear mixed effect model using a Tukey-Kramer HSD post-hoc analysis. Asterisks indicate a p-value < 0.05. All ELISAs were repeated two times.
(TIF)

S7 Fig Anti-uRBC IgG and IgM are inversely correlated with hemoglobin levels during primary *P. cynomolgi* infections. Anti-uRBC and anti-iRBC antibody response for IgM (a) and IgG (b) during primary infections and relapses as determined by ELISA. Dashed and solid lines indicate background levels as defined by the mean of the malaria naïve samples plus three standard deviations for uninfected and infected RBCs, respectively. Spearman’s correlation analysis of anti-uRBC IgM (c) and anti-uRBC IgG (d) with hemoglobin levels during primary and relapse infections. Bars indicate the mean of the data points shown; Error Bars = SEM. Statistical significance was assessed by a linear mixed effect model using a Tukey-Kramer HSD post-hoc analysis. Asterisks indicate a p-value < 0.05. All ELISAs were repeated two times.
(TIF)

S8 Fig Pre-existing immunity does not change day-to-patency during homologous challenges. The days to patency for the initial infections and homologous challenges are shown. Statistical significance was assessed by a Wilcoxon test.
(TIF)

S9 Fig Hemoglobin and platelet kinetics with parasitemia during homologous challenges with *P. cynomolgi* M/B strain in rhesus macaques. Daily hemoglobin levels (a) and platelet numbers (b) during homologous challenges with *P*. *cynomolgi* M/B strain. The five-letter code on the left-hand side of each graph indicates a different individual. k = multiply number shown by 1,000.
(TIF)

S10 Fig Spearman’s correlation analysis of the relationship between naïve and double negative B cells during relapses and homologous reinfections. ρ = Spearman’s correlation coefficient.
(TIF)

S11 Fig Total IgM, total IgG, and IgG subclass kinetics during *P. cynomolgi* relapses and homologous reinfections. Kinetics of total IgM (a) and IgG (b) at different infection stages during relapses and homologous reinfections as determined by ELISA. (c) IgG subclasses recognizing iRBC lysates as determined by ELISAs. The black line indicates the background. Pink diamonds = malaria naïve, orange circles = initial infections, and green triangles = relapse infections. Bars indicate the mean of the data points shown; Error Bars = SEM. Statistical significance was assessed by a linear mixed effect model using a Tukey-Kramer HSD post-hoc analysis. Asterisks indicate a p-value < 0.05. All ELISAs were repeated two times.
(TIF)

S12 Fig Anti-uRBC IgM and IgG kinetics during relapses and homologous reinfections. Anti-uRBC and anti-iRBC antibody response for IgM (a) and IgG (b) during relapses and homologous reinfections as determined by ELISA. Dashed and solid lines indicate background levels indicate a true positive as defined by the mean of the malaria naïve samples plus three standard deviations for uninfected and infected RBCs, respectively. Bars indicate the mean of the data points shown; Error Bars = SEM. Statistical significance was assessed by a linear mixed effect model using a Tukey-Kramer HSD post-hoc analysis. Asterisks indicate a p-value < 0.05. All ELISAs were repeated two times.
(TIF)

S1 Table Collection criteria for MaHPIC Experiment 23 time points. (XLSX)

S2 Table Pathways enrichment analysis of upregulated genes during relapses. (XLSX)

S3 Table Pathways enrichment analysis of downregulated genes during relapses. (XLSX)

S4 Table Collection criteria for MaHPIC Experiment 24 time points. (XLSX)

S5 Table Pathway enrichment analysis of upregulated genes during homologous reinfections. (XLSX)

S6 Table Normalized gene expression table for *Macaca mulatta* transcripts during Experiment 23 and Experiment 24. (XLSX)

S7 Table Normalized gene expression table for Experiment 23 *Plasmodium cynomolgi* B/M strain gene expression. (XLSX)

S8 Table Flow cytometry panels used for monitoring B cell subsets during primary and relapse infections. (XLSX)

S9 Table Flow cytometry panels used for monitoring B cell subsets during homologous reinfections. (XLSX)
