# Supplementary figures and images for: Humoral immunity prevents clinical malaria during Plasmodium relapses without eliminating gametocytes
Source: PLoS Pathog. 2019 Sep 19;15(9):e1007974. doi: 10.1371/journal.ppat.1007974 (PMC6752766; doi:10.1371/journal.ppat.1007974)

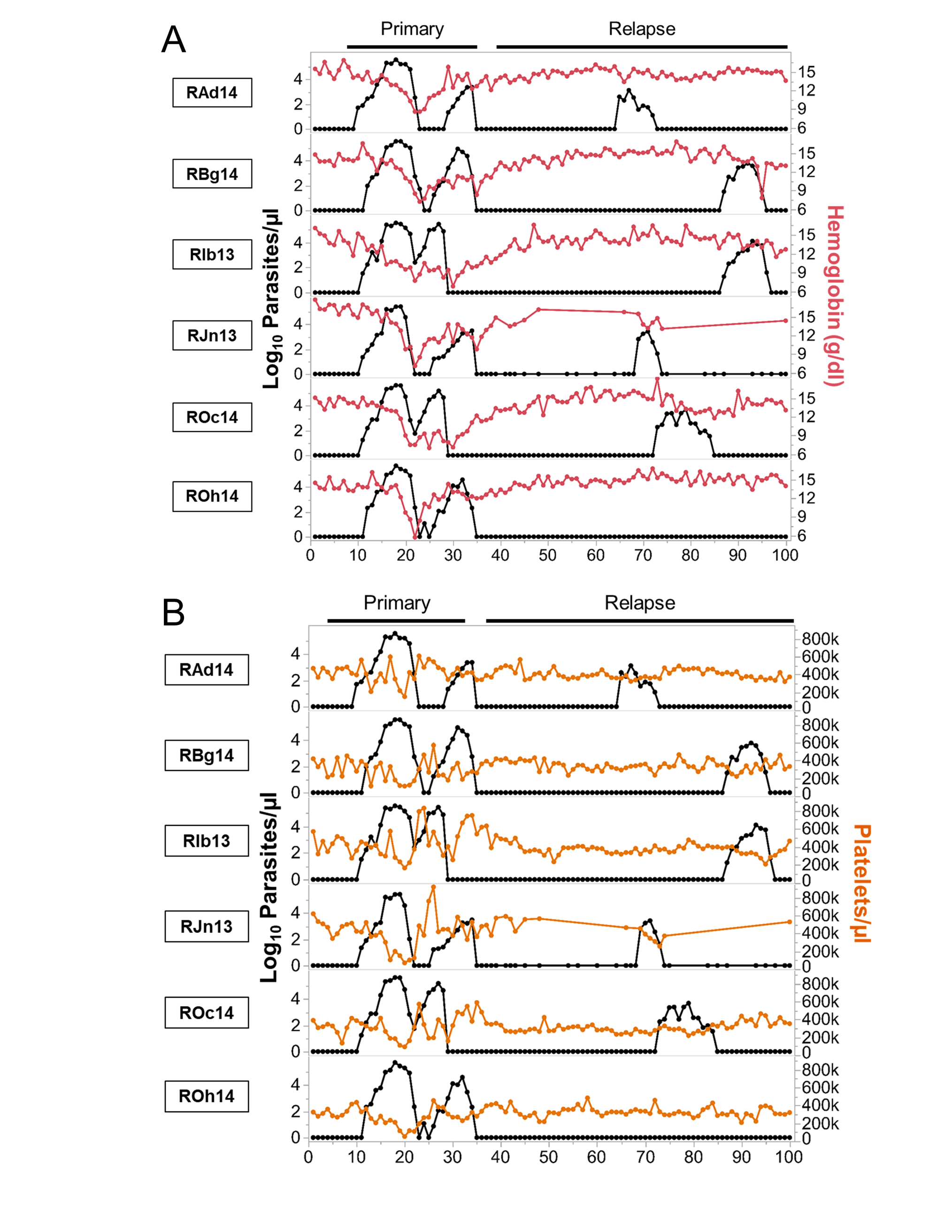

Supplement: S1 Fig — Daily hemoglobin levels (a) and platelet numbers (b) during initial and relapsing P. cynomolgi M/B strain infections. The five-letter code on the left-hand side of each graph indicates a different individual rhesus macaques. k = multiply number shown by 1,000. (TIF) [file ppat.1007974.s001.tif]

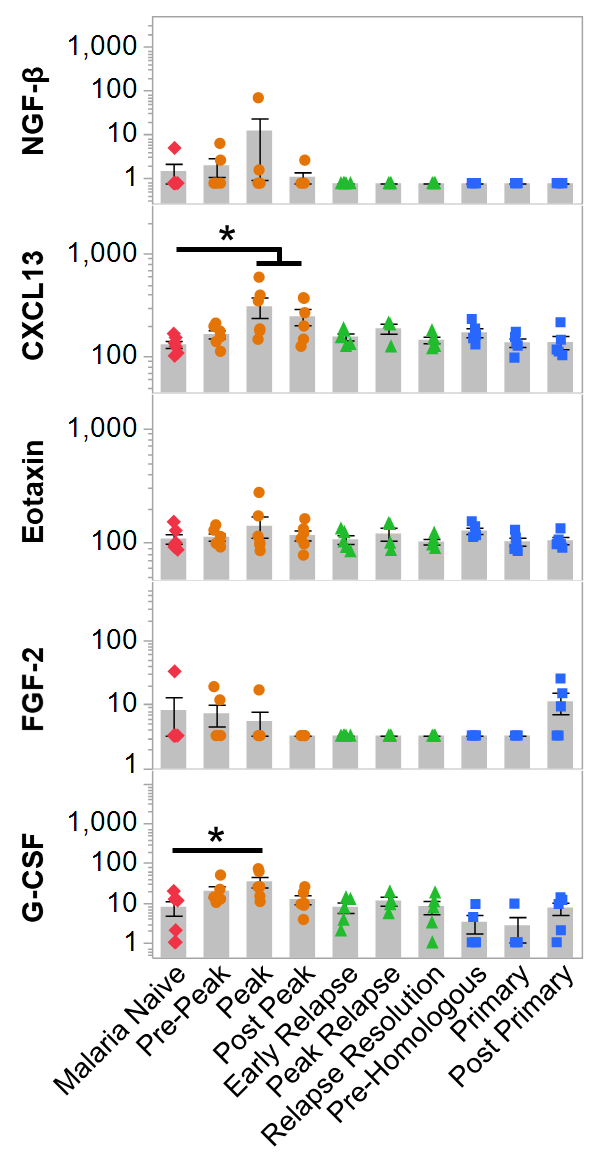


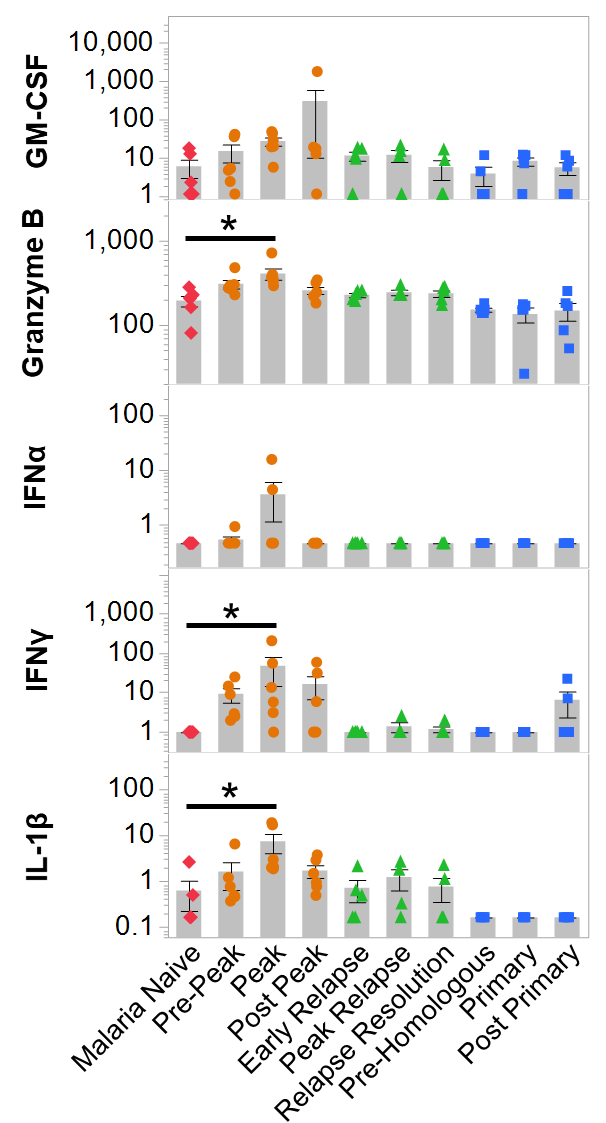


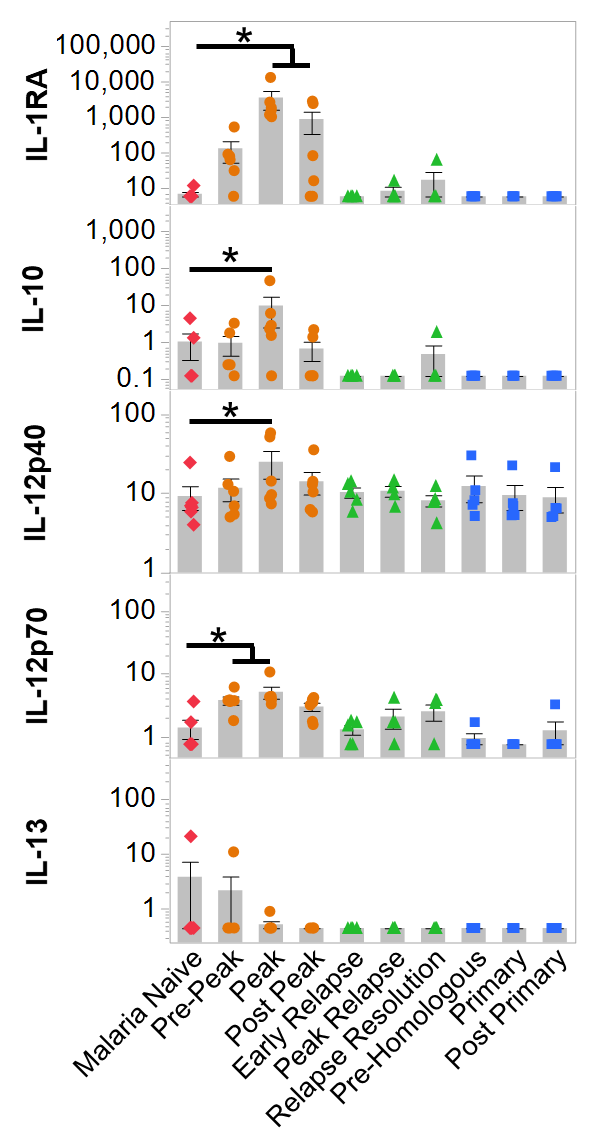


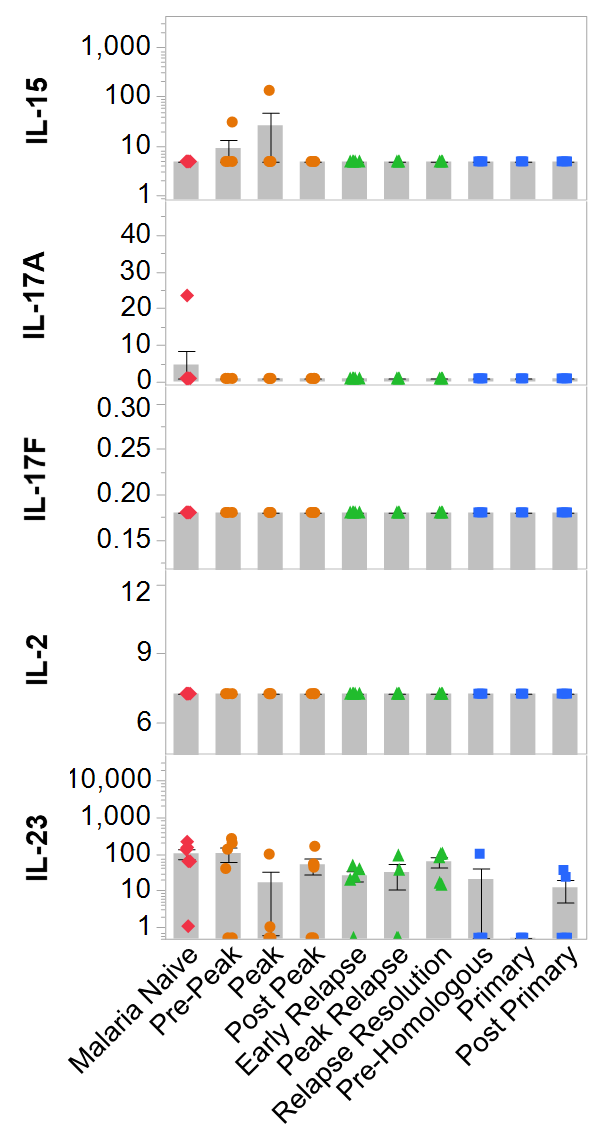


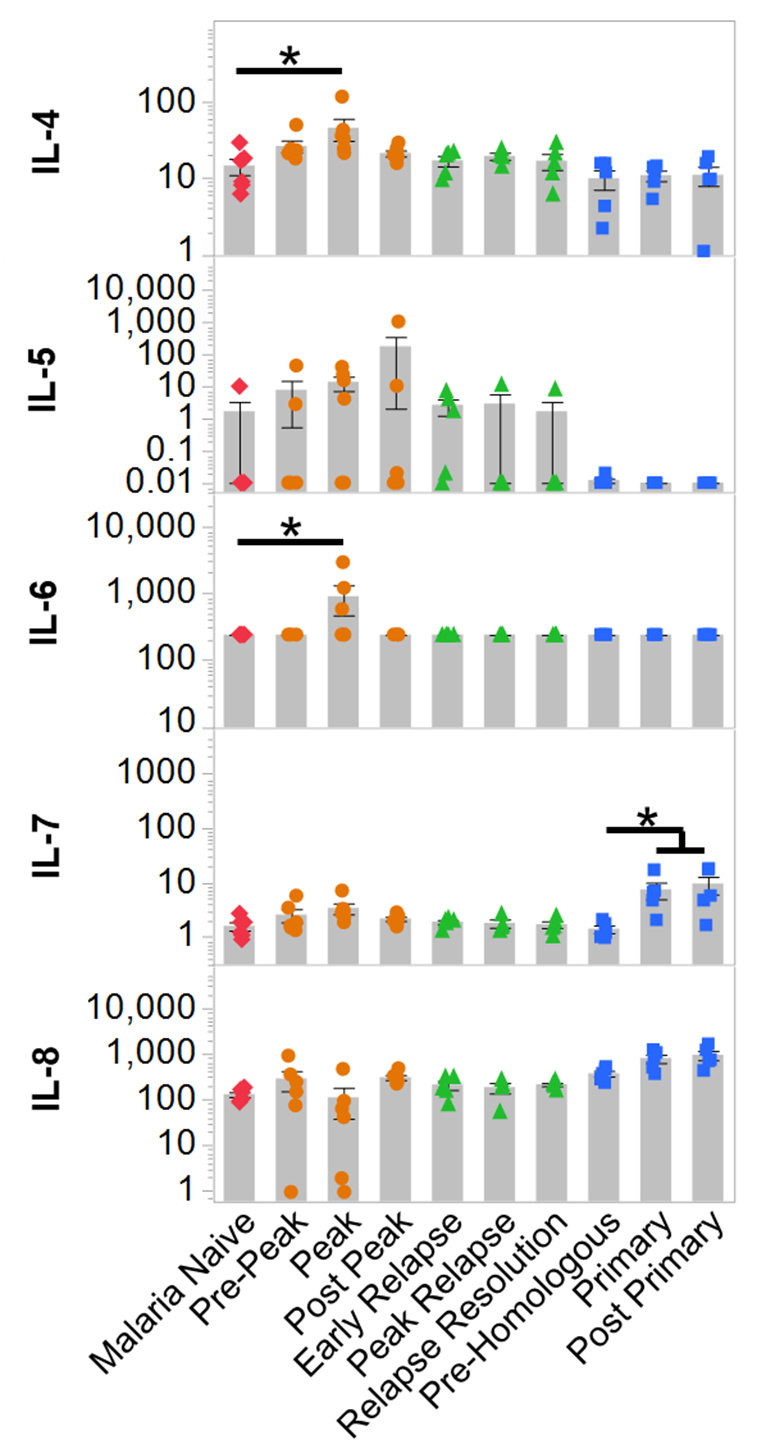


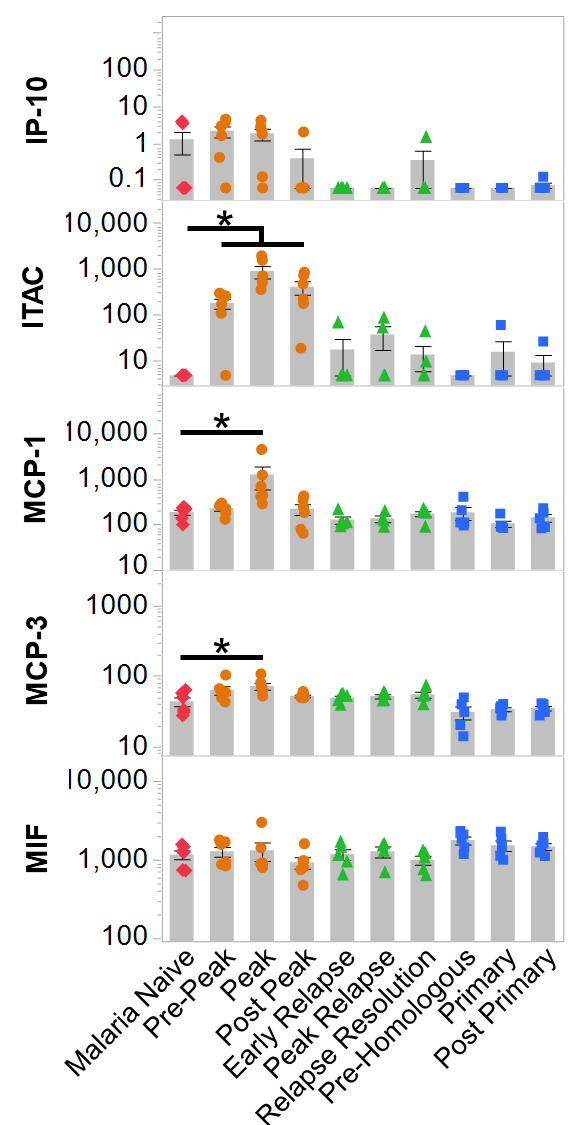


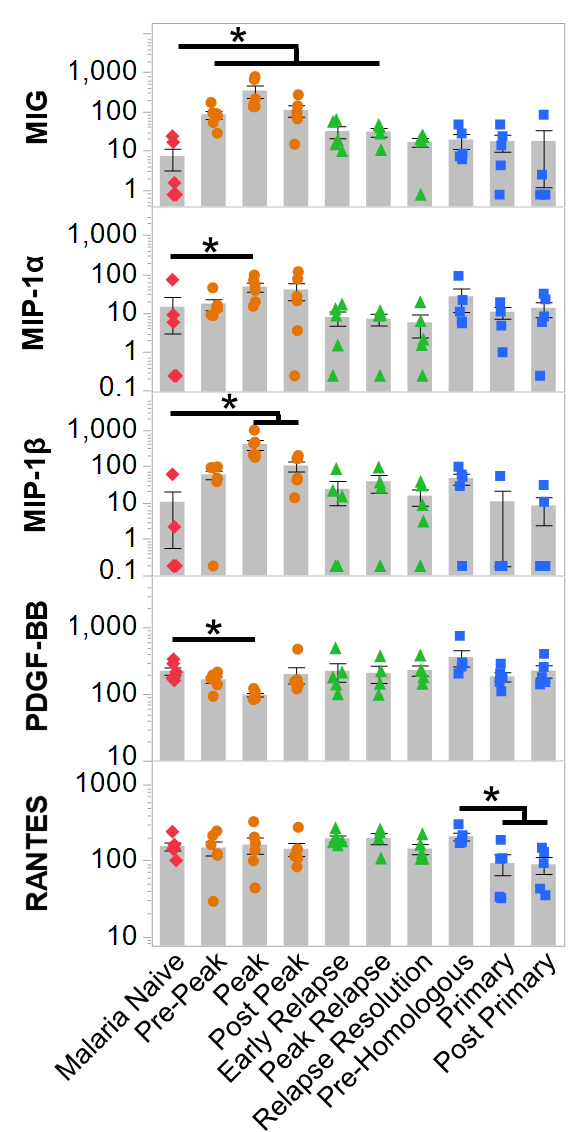


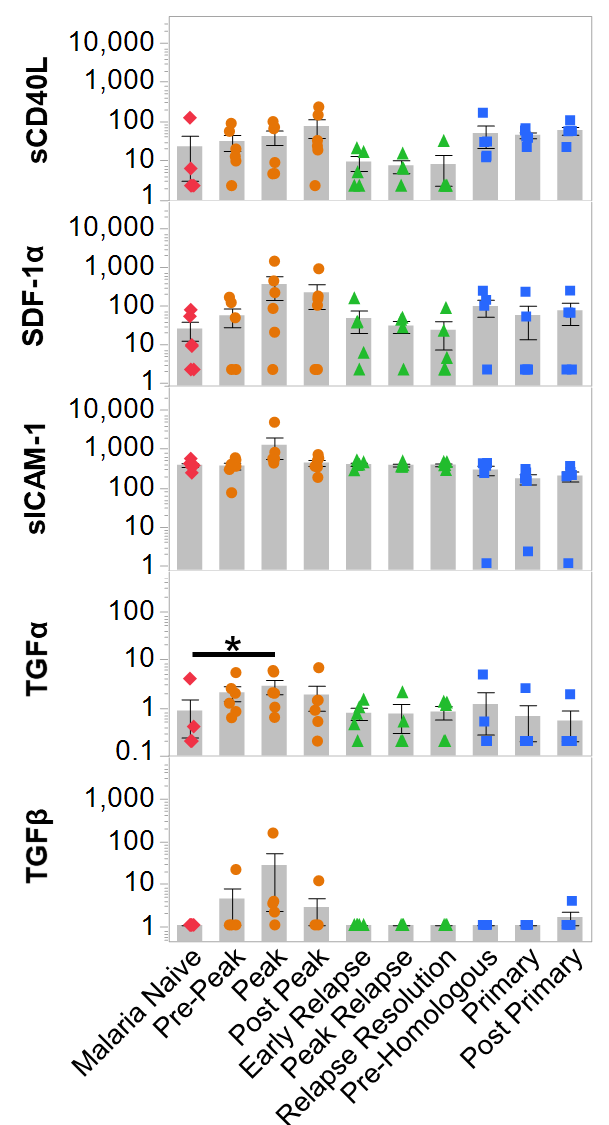


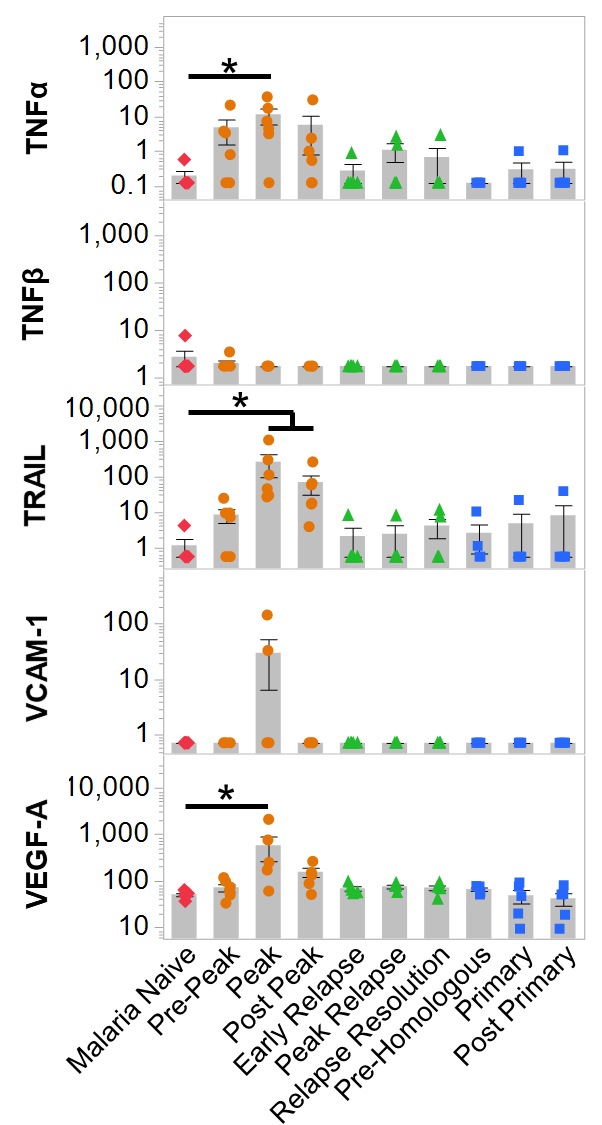

Supplement: S2 Fig — Statistical significance was assessed by a linear mixed effect model using a Tukey-Kramer HSD post-hoc analysis. Asterisks indicate a p-value < 0.05. (DOCX) [file ppat.1007974.s002.docx]

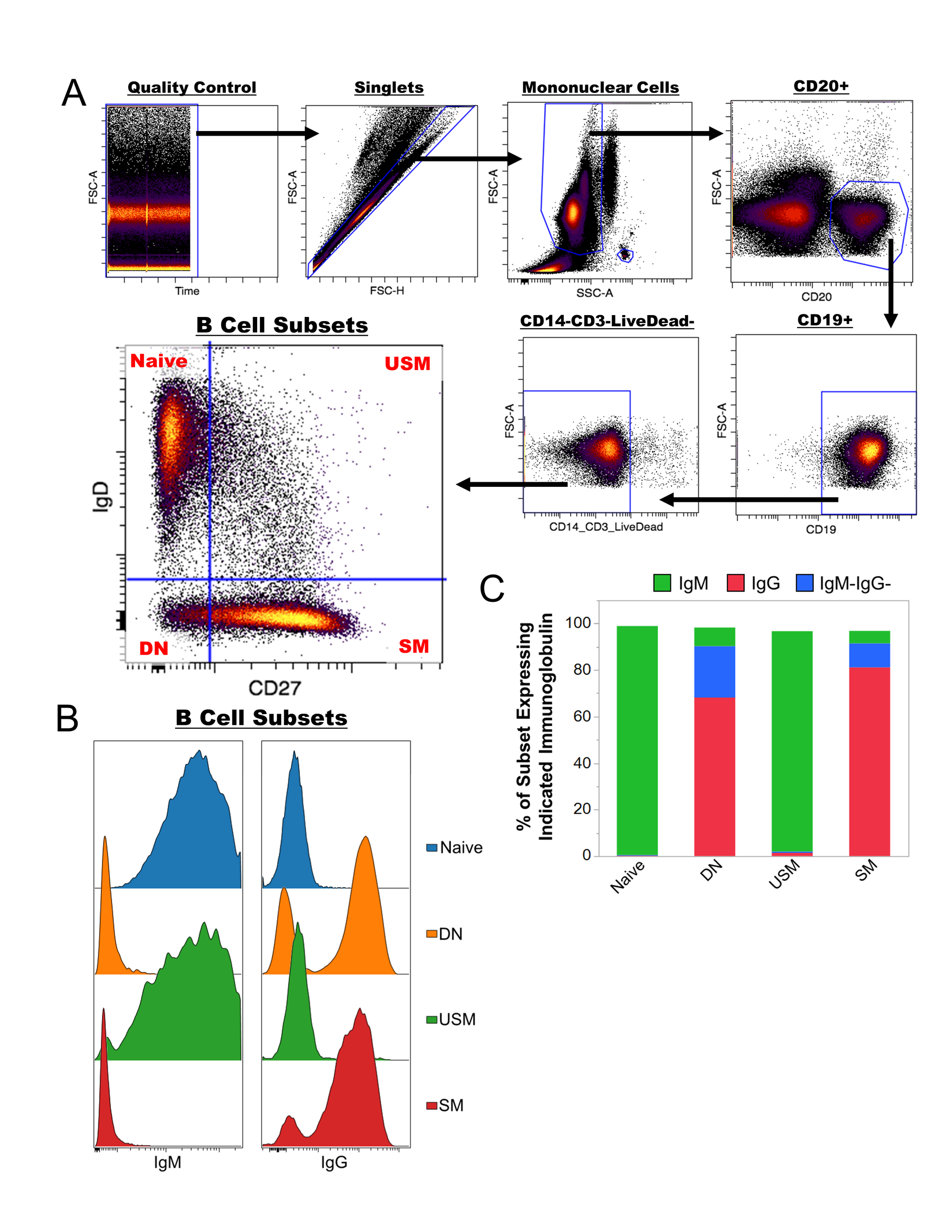

Supplement: S3 Fig — (a) A representative gating strategy for monitoring rhesus macaque B cell subsets in PBMCs is shown. (b) Histograms of surface IgM and IgG expression of the four B cell subsets being monitored from the representative sample shown in panel a. (c) The average percentage of each B cell subset that are IgG+, IgM+, or IgM-IgG-USM from six malaria naïve rhesus macaques. USM = Unswitched Memory, DN = Double-Negative, SM = Switched Memory. (TIF) [file ppat.1007974.s003.tif]

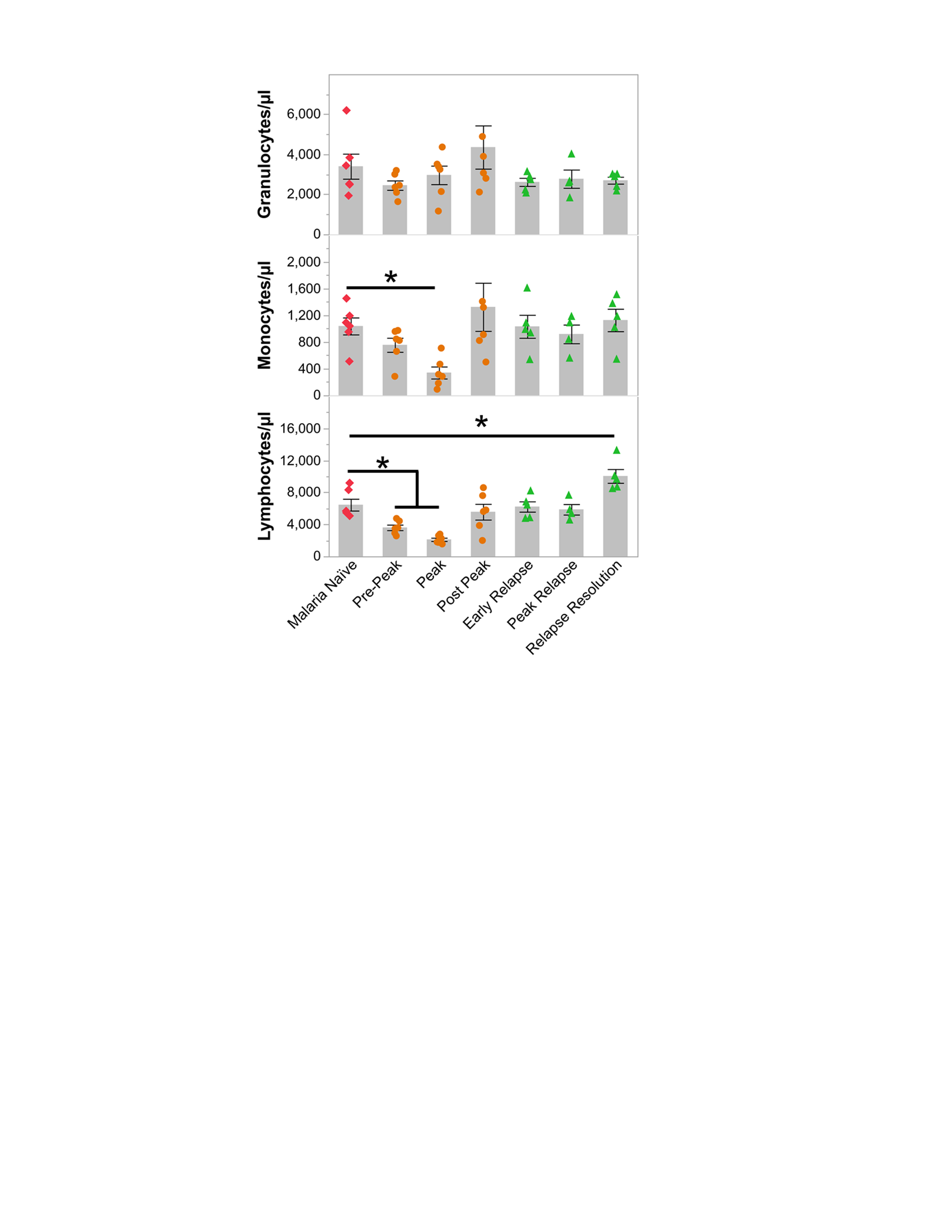

Supplement: S4 Fig — The absolute number of granulocytes, monocytes, and lymphocytes per microliter of blood during initial infections and relapses as determined by complete blood counts. Pink diamonds = malaria naïve, orange circles = initial infection, and green triangles = relapse infection. Gray bars indicate the mean of the data points shown; Error Bars = SEM. Statistical significance was assessed by a linear mixed effect model using a Tukey-Kramer HSD post-hoc analysis. Asterisks indicate a p-value < 0.05. (TIF) [file ppat.1007974.s004.tif]

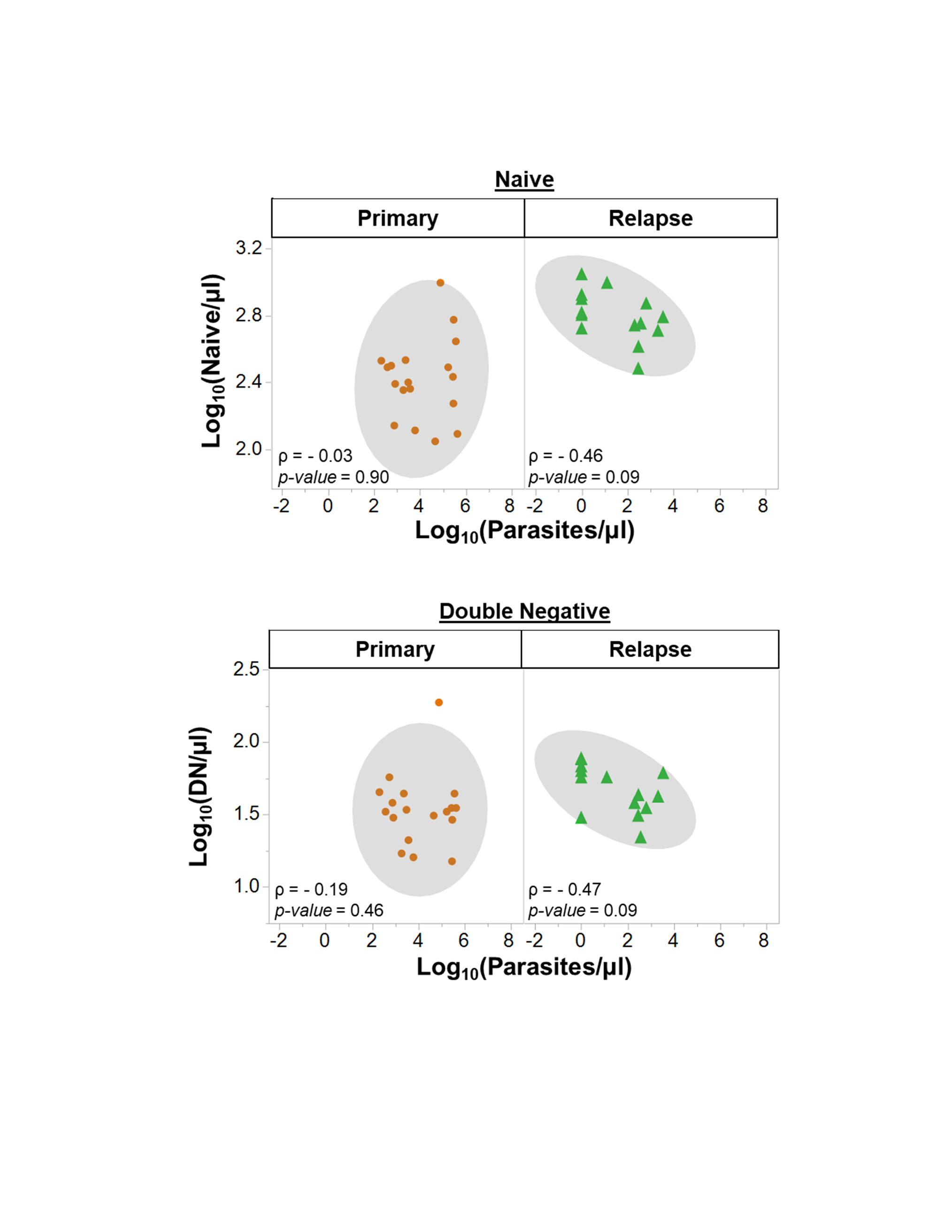

Supplement: S5 Fig — ρ = Spearman’s correlation coefficient. (TIF) [file ppat.1007974.s005.tif]

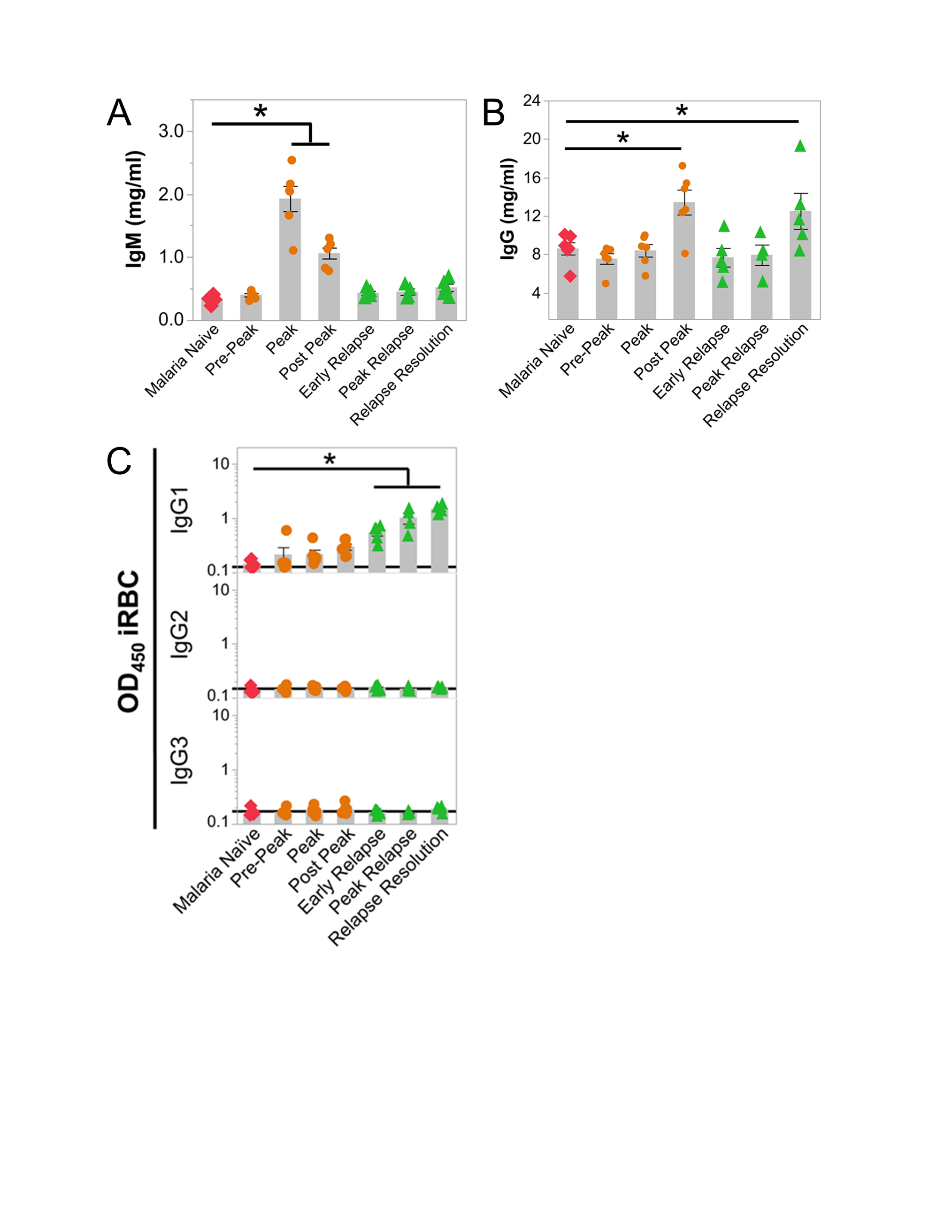

Supplement: S6 Fig — Kinetics of total IgM (a) and IgG (b) at different infection stages during initial infections and relapses as determined by ELISA. (c) IgG subclasses recognizing iRBC lysates as determined by ELISAs. The black line indicates background. Pink diamonds = malaria naïve, orange circles = initial infections, and green triangles = relapse infections. Bars indicate the mean of the data points shown; Error Bars = SEM. Statistical significance was assessed by a linear mixed effect model using a Tukey-Kramer HSD post-hoc analysis. Asterisks indicate a p-value < 0.05. All ELISAs were repeated two times. (TIF) [file ppat.1007974.s006.tif]

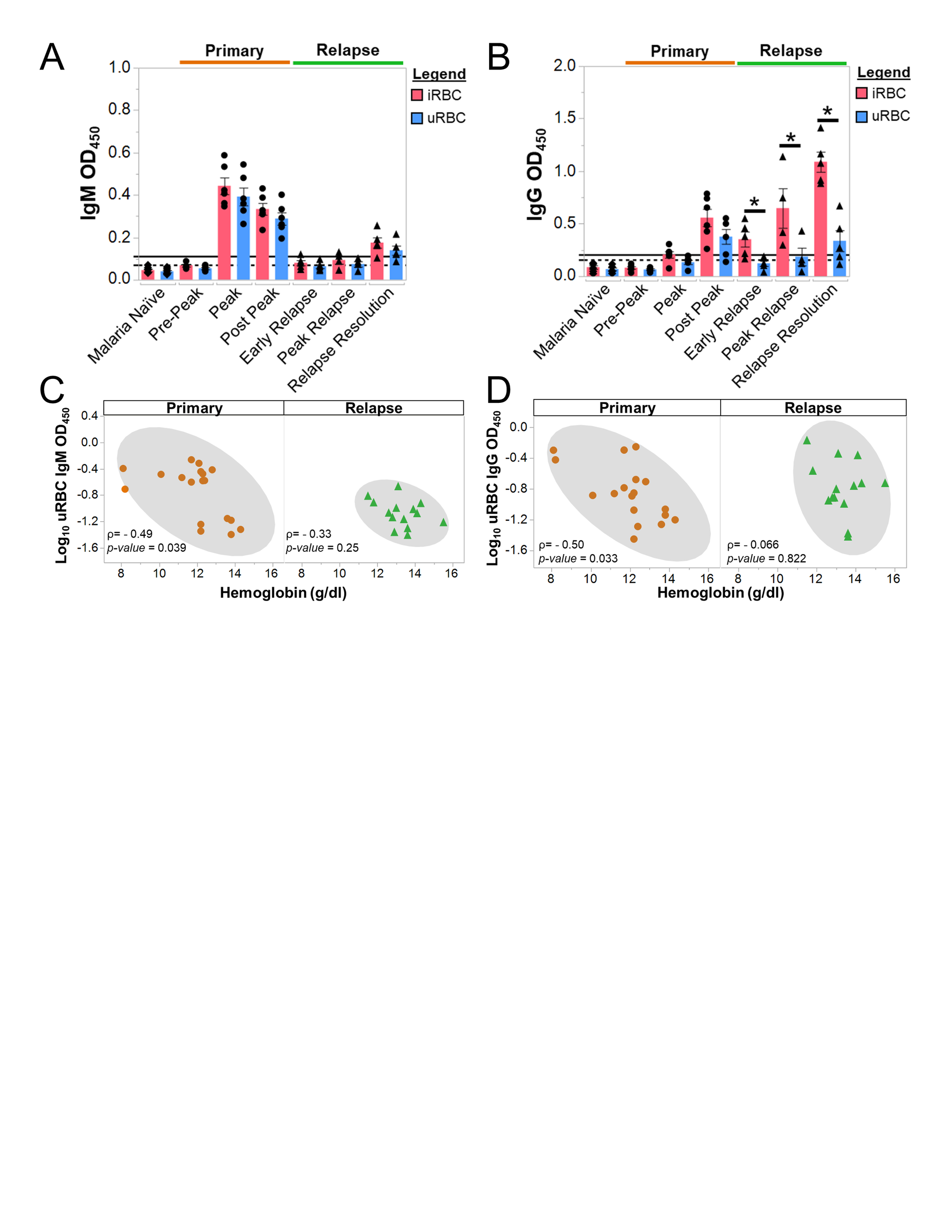

Supplement: S7 Fig — Anti-uRBC and anti-iRBC antibody response for IgM (a) and IgG (b) during primary infections and relapses as determined by ELISA. Dashed and solid lines indicate background levels as defined by the mean of the malaria naïve samples plus three standard deviations for uninfected and infected RBCs, respectively. Spearman’s correlation analysis of anti-uRBC IgM (c) and anti-uRBC IgG (d) with hemoglobin levels during primary and relapse infections. Bars indicate the mean of the data points shown; Error Bars = SEM. Statistical significance was assessed by a linear mixed effect model using a Tukey-Kramer HSD post-hoc analysis. Asterisks indicate a p-value < 0.05. All ELISAs were repeated two times. (TIF) [file ppat.1007974.s007.tif]

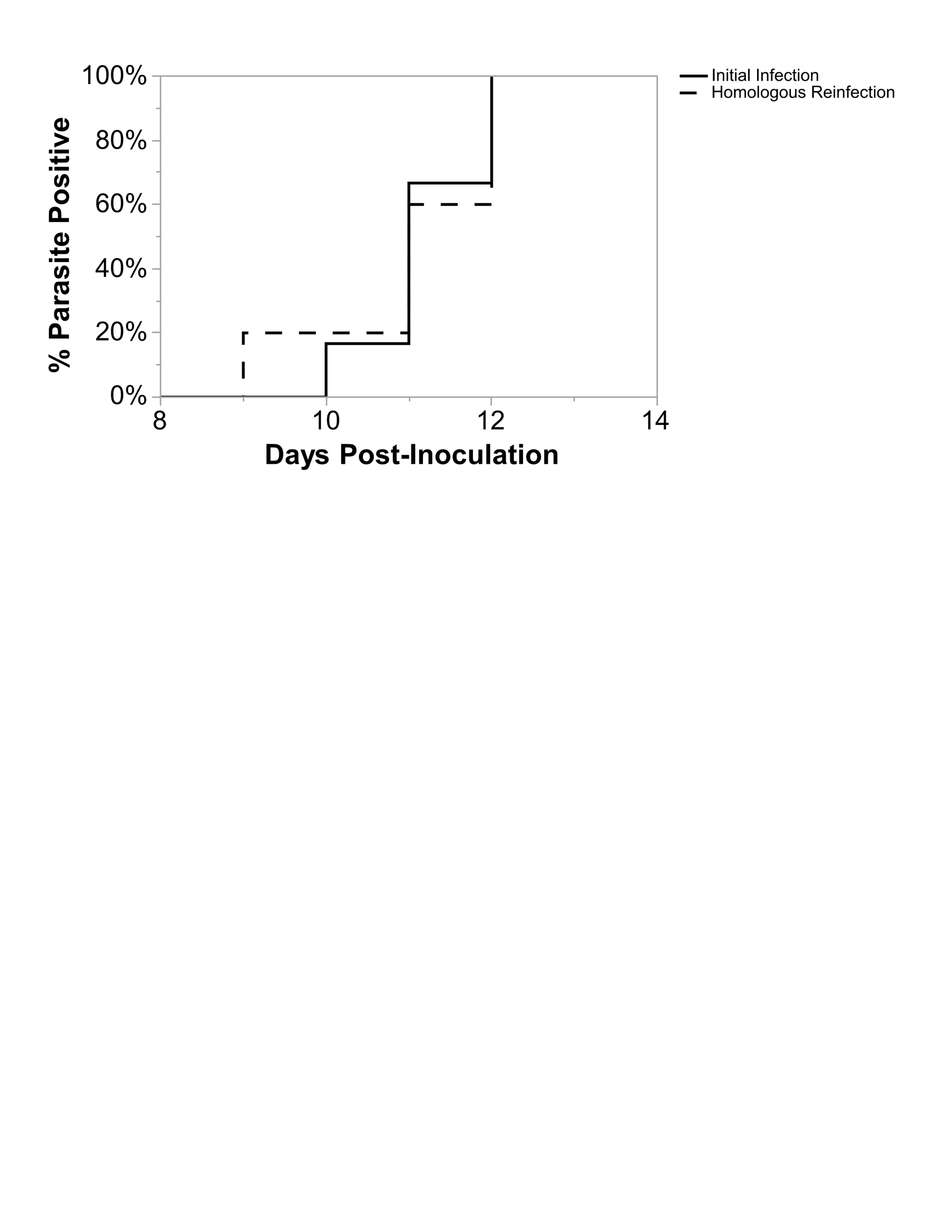

Supplement: S8 Fig — The days to patency for the initial infections and homologous challenges are shown. Statistical significance was assessed by a Wilcoxon test. (TIF) [file ppat.1007974.s008.tif]

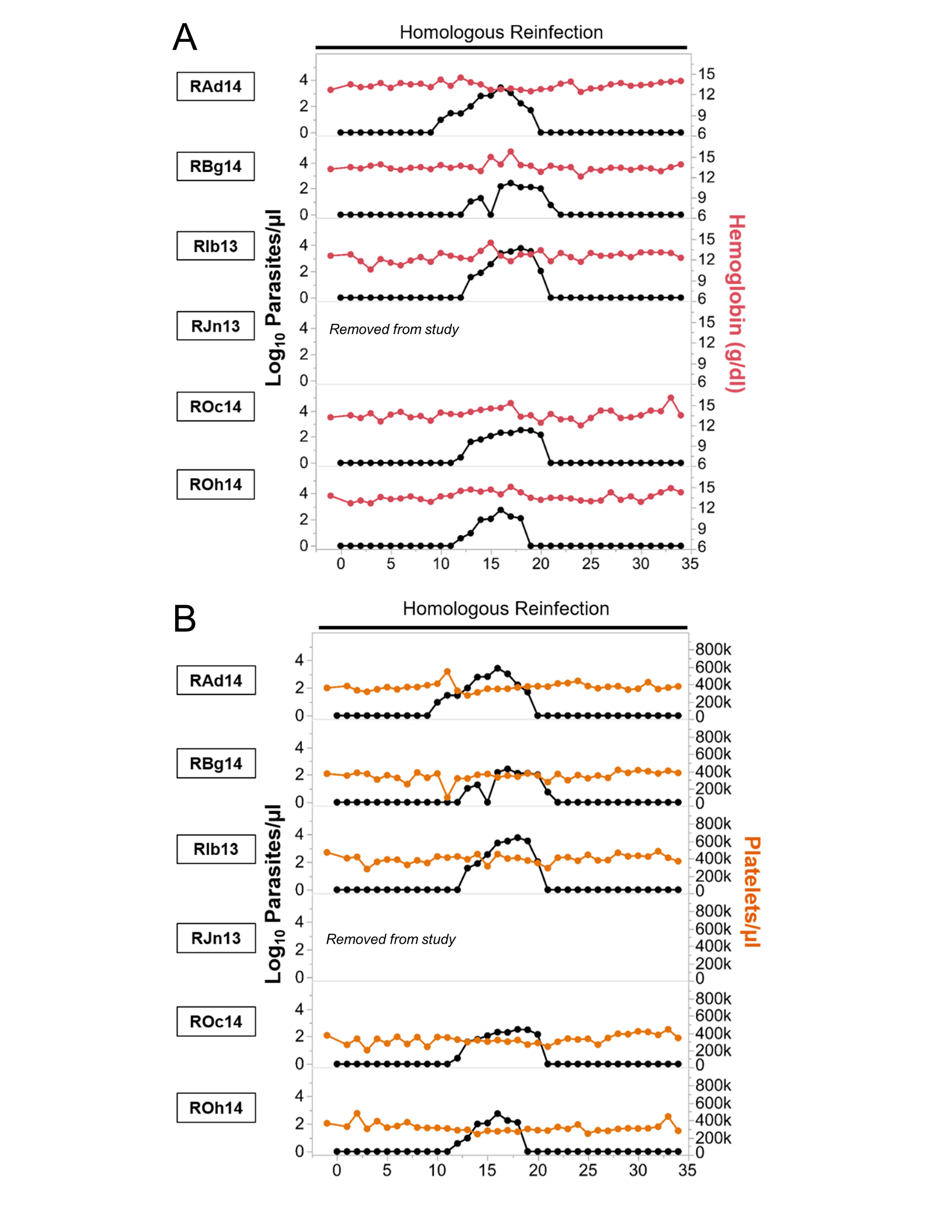

Supplement: S9 Fig — Daily hemoglobin levels (a) and platelet numbers (b) during homologous challenges with P. cynomolgi M/B strain. The five-letter code on the left-hand side of each graph indicates a different individual. k = multiply number shown by 1,000. (TIF) [file ppat.1007974.s009.tif]

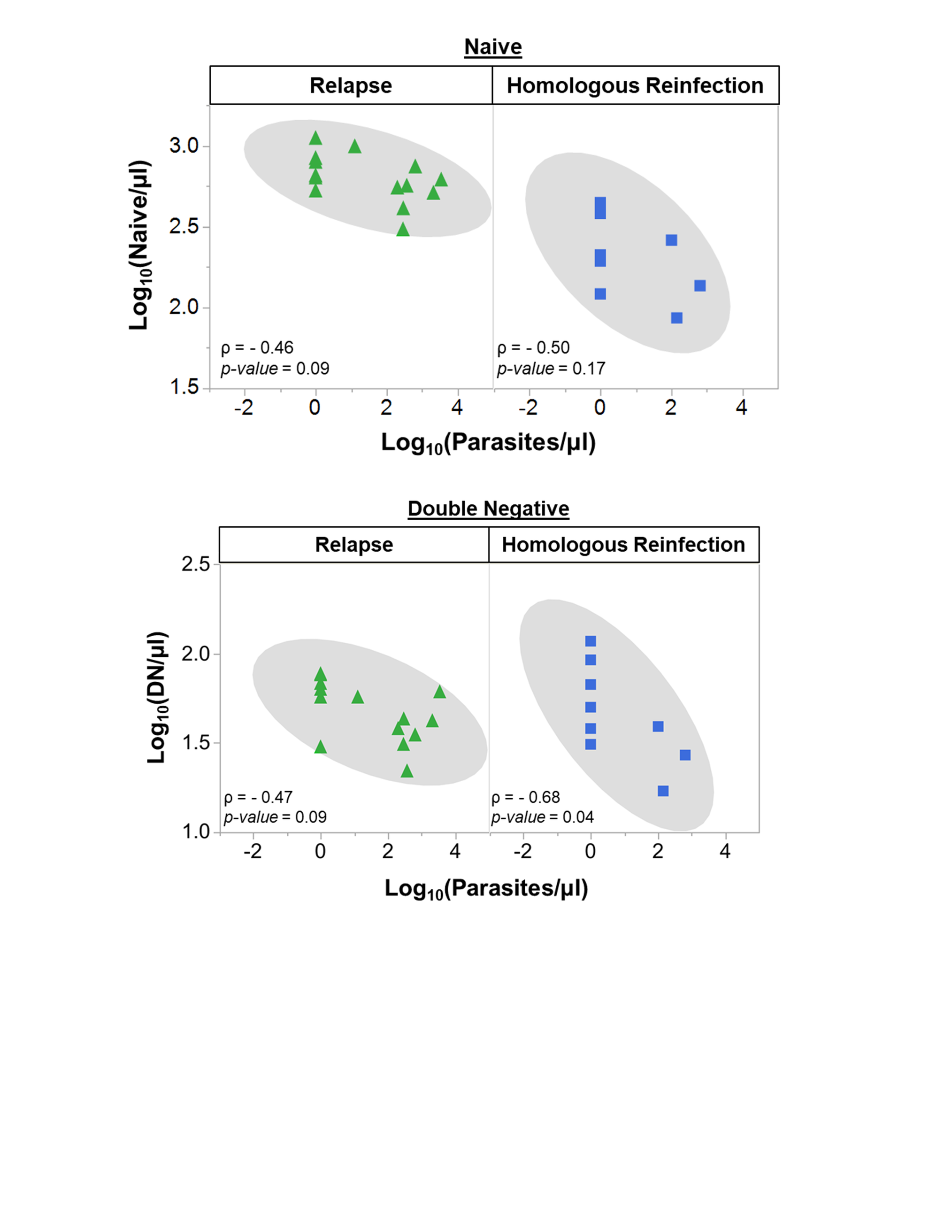

Supplement: S10 Fig — ρ = Spearman’s correlation coefficient. (TIF) [file ppat.1007974.s010.tif]

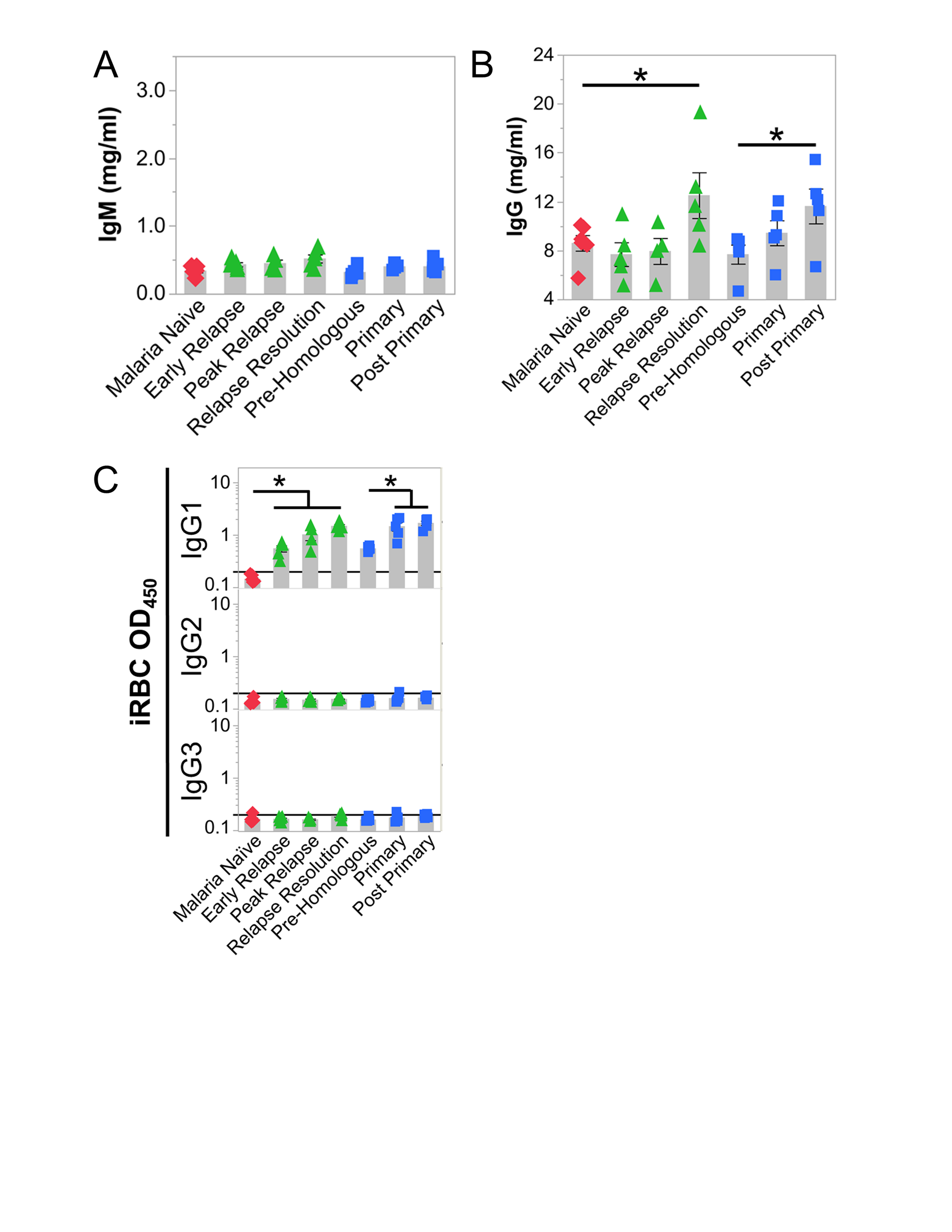

Supplement: S11 Fig — Kinetics of total IgM (a) and IgG (b) at different infection stages during relapses and homologous reinfections as determined by ELISA. (c) IgG subclasses recognizing iRBC lysates as determined by ELISAs. The black line indicates the background. Pink diamonds = malaria naïve, orange circles = initial infections, and green triangles = relapse infections. Bars indicate the mean of the data points shown; Error Bars = SEM. Statistical significance was assessed by a linear mixed effect model using a Tukey-Kramer HSD post-hoc analysis. Asterisks indicate a p-value < 0.05. All ELISAs were repeated two times. (TIF) [file ppat.1007974.s011.tif]

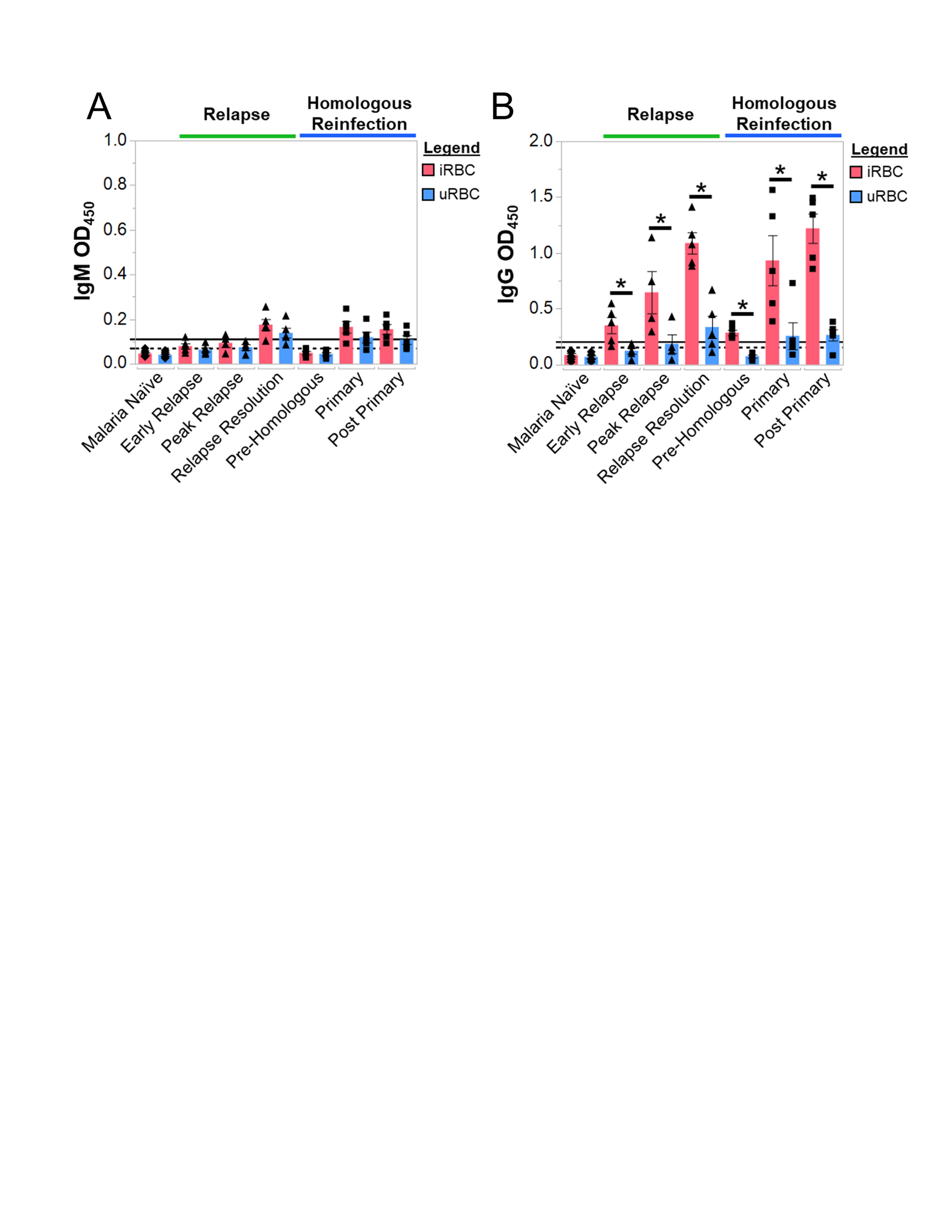

Supplement: S12 Fig — Anti-uRBC and anti-iRBC antibody response for IgM (a) and IgG (b) during relapses and homologous reinfections as determined by ELISA. Dashed and solid lines indicate background levels indicate a true positive as defined by the mean of the malaria naïve samples plus three standard deviations for uninfected and infected RBCs, respectively. Bars indicate the mean of the data points shown; Error Bars = SEM. Statistical significance was assessed by a linear mixed effect model using a Tukey-Kramer HSD post-hoc analysis. Asterisks indicate a p-value < 0.05. All ELISAs were repeated two times. (TIF) [file ppat.1007974.s012.tif]
